# The Identification of Gut Neuroendocrine Tumor Disease by Multiple Synchronous Transcript Analysis in Blood

**DOI:** 10.1371/journal.pone.0063364

**Published:** 2013-05-15

**Authors:** Irvin M. Modlin, Ignat Drozdov, Mark Kidd

**Affiliations:** Department of Surgery, Yale University School of Medicine, New Haven, Connecticut, United States of America; Peking Union Medical College Hospital, Peking Union Medical College, Chinese Academy of Medical Sciences, China

## Abstract

Gastroenteropancreatic (GEP) neuroendocrine neoplasms (NENs) are increasing in both incidence and prevalence. A delay in correct diagnosis is common for these lesions. This reflects the absence of specific blood biomarkers to detect NENs. Measurement of the neuroendocrine secretory peptide Chromogranin A (CgA) is used, but is a single value, is non-specific and assay data are highly variable. To facilitate tumor detection, we developed a multi-transcript molecular signature for PCR-based blood analysis. NEN transcripts were identified by computational analysis of 3 microarray datasets: NEN tissue (*n* = 15), NEN peripheral blood (*n* = 7), and adenocarcinoma (*n* = 363 tumors). The candidate gene signature was examined in 130 blood samples (NENs: *n* = 63) and validated in two independent sets (Set 1 [*n* = 115, NENs: *n* = 72]; Set 2 [*n* = 120, NENs: *n* = 58]). Comparison with CgA (ELISA) was undertaken in 176 samples (NENs: *n* = 81). 51 significantly elevated transcript markers were identified. Gene-based classifiers detected NENs in independent sets with high sensitivity (85–98%), specificity (93–97%), PPV (95–96%) and NPV (87–98%). The AUC for the NEN gene-based classifiers was 0.95–0.98 compared to 0.64 for CgA (Z-statistic 6.97–11.42, *p*<0.0001). Overall, the gene-based classifier was significantly (χ^2^ = 12.3, *p*<0.0005) more accurate than CgA. In a sub-analysis, pancreatic NENs and gastrointestinal NENs could be identified with similar efficacy (79–88% sensitivity, 94% specificity), as could metastases (85%). In patients with low CgA, 91% exhibited elevated transcript markers. A panel of 51 marker genes differentiates NENs from controls with a high PPV and NPV (>90%), identifies pancreatic and gastrointestinal NENs with similar efficacy, and confirms GEP-NENs when CgA levels are low. The panel is significantly more accurate than the CgA assay. This reflects its utility to identify multiple diverse biological components of NENs. Application of this sensitive and specific PCR-based blood test to NENs will allow accurate detection of disease, and potentially define disease progress enabling monitoring of treatment efficacy.

## Introduction

Although previously considered rare, gastroenteropancreatic neuroendocrine neoplasms (GEP-NENs) are common (incidence: 3.6/100,000), occurring as frequently as testicular tumors, Hodgkin’s disease, gliomas and multiple myeloma [1] and are estimated to have a prevalence of 35/100,000 [2]. They represent a significant clinical issue since 50–70% are metastatic at diagnosis and there is a paucity of effective therapy. Two common agents, everolimus and sunitinib, only increase progression free survival by ∼6 months, while somatostatin analogs have a marginal impact. The lack of sensitive and robust biomarkers to establish diagnosis, assess disease progress and monitor treatment efficacy has been identified as key unmet needs [3].

Strategies including staging at surgery, pathological grading, blood Chromogranin A (CgA) measurements, detection of circulating tumor cells (CTCs) or other products e.g. serotonin are currently used [1]. Their utility is highly variable and often insensitive for small tumors or metastasis detection, may require tissue and depends on non-standardized tests. Despite that CgA has been proposed as a marker of disease and tool for evaluating treatment efficacy [4], it is not FDA-accepted as a supportable biomarker [5]. This reflects limitations in sensitivity, specificity and reproducibility.

Identification of a peripherally accessible, molecular fingerprint using PCR-amplification of target genes, has successfully been undertaken in other cancers e.g., breast and colon. In the former, this is used in prognosis, identification of metastasis and recurrence, prediction of therapy response and metastasis-free survival for node-negative, untreated primary cancers [6], [7]; for the latter, utility has been determined for staging [8]. We report the initial assessment of our hypothesis that a neoplasia-associated circulating signature is identifiable in GEP-NENs and can be used to accurately identify disease. We have previously evaluated tissue-derived gene markers for GEP-NENs [9]–[11] and demonstrated their utility for detecting NEN malignancy [12]. In this study, we extended this strategy developing a blood-based PCR test using REMARK (REporting of tumor MARKer studies) criteria [13] to detect circulating mRNAs that facilitate GEP-NEN diagnosis and management.

## Materials and Methods

Detailed methods are available in the online supplement including computational analyses, collection methodology, sampling and handling. All samples were collected and analyzed according to an IRB protocol (Yale University School of Medicine). The protocol was specifically approved for this study. Written consent was obtained from all study participants.

### In silico Identification of 51 Marker Genes

Human cancer and normal tissue microarray datasets were obtained (ArrayExpress database [14]). Two GEP-NEN gene expression datasets were analyzed (GEP-NEN-A, GEP-NEN-B). The former included small intestinal tissue (*n* = 3; macroscopically normal mucosa collected at surgery), primary GEP-NENs (*n* = 6), and metastatic GEP-NENs (*n* = 3) [15]; the latter, normal ileal mucosa (*n* = 6), primary midgut NENs (*n* = 3), and liver metastases (*n* = 3) [16]. Additionally, a compendium of public cancer microarray datasets (three hepatocellular carcinoma (HCC) datasets [Alcohol-HCC (*n* = 65 arrays), Viral-HCC (*n* = 124 arrays), and Progression-HCC (*n* = 75 arrays)]; breast (*n* = 86 arrays, colon (*n* = 47 arrays, and prostate (*n* = 154 arrays) cancer profiles and normal human tissue arrays (*n* = 158 arrays)] were analyzed (**Table S1**).

Thereafter, we examined gene expression in peripheral blood. For this, fourteen samples (controls: *n* = 7; GEP-NENs: *n* = 7) were examined. Samples were processed using the Applied Biosystems Tempus Spin RNA Isolation t Kit (RNA quality >1.8 A_260∶280_ ratio, RIN>7.0). RNA was hybridized on Affymetrix platforms [11], [12] and analysis performed as described previously [17].

**Figure 1:**
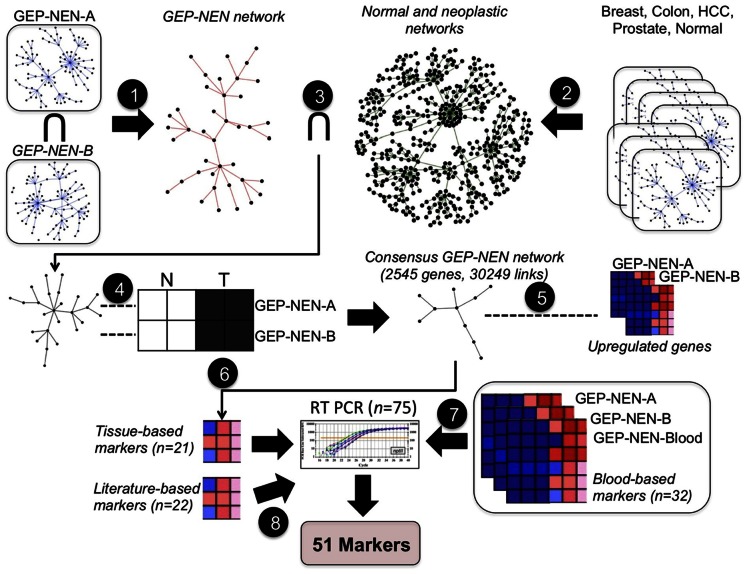
Computational pipeline used to derive a set of 51 markers that identify GEP-NEN disease. **Step 1:** Gene co-expression networks inferred from GEP-NEN-A and GEP-NEN-B datasets are intersected, producing the GEP-NEN network. **Step 2:** Co-expression networks from neoplastic and normal tissue microarray datasets are combined to produce the normal and neoplastic networks. **Step 3:** Links present in normal and neoplastic networks are subtracted from the GEP-NEN network. **Step 4:** Concordantly regulated genes in GEP-NEN-A and GEP-NEN-B networks are retained; other genes are eliminated from the GEP-NEN network, producing the Consensus GEP-NEN network. **Step 5:** Upregulated genes in both the GEP-NEN-A and GEP-NEN-B dataset are mapped to the Consensus GEP-NEN network. **Step 6:** Topological filtering, expression profiling, and literature-curation of putative tissue-based markers, yielding 21 putative genes further examined by RT-PCR. **Step 7:** Identification of mutually up-regulated genes in GEP-NEN blood transcriptome and GEP-NEN-A and GEP-NEN-B datasets, yielding 32 putative genes further examined by RT-PCR. **Step 8:** Literature-curation and cancer mutation database search, yielding a panel of 22 putative marker genes for further RT-PCR analysis.

Details of the microarray analyses and gene identification including normalization using Robust Multi-array Average (RMA) [18], identifying probes and mapping to Ensembl gene identifiers, assessment of gene co-expression network inferences, network partitioning and functional enrichment analyses [19] are included in the Supplemental methods. This computational approach (**Figure 1** **, Figure S2**) resulted in the identification of 75 candidate genes. Preliminary screening detected 51 marker genes which were then included in the current study.

### RNA Isolation and cDNA Synthesis from Peripheral Blood

*Training set*: Transcripts (mRNA) were isolated from 130 blood samples (controls: *n* = 67; GEP-NENs: *n* = 63) using the mini blood kit (Qiagen: RNA quality >1.8 A_260∶280_ ratio, RIN>5.0) with cDNA produced using the High Capacity Reverse transcriptase kit (Applied Biosystems: cDNA production 2000–2500ng/ul).

*Independent Validation sets*: Two sets were used: the first included 115 samples (controls: *n* = 43; GEP-NENs: *n* = 72), and the second, 120 samples (controls: *n* = 49; GEP-NENs: *n* = 71) (**Table 1**). The clinical characteristics are included in **Table 2**. Small intestinal and pancreatic tumors (67–86%) were predominant, lesions were grade 1 and 2 (Ki67≤20%) (76–89%) and metastases (73–85%) were prevalent. Patients were older than controls, and sex-matching was not undertaken.

**Table 1:** Characteristics of patient and controls (training and independent sets).

Characteristic	Cases	Controls	p-Value
Training Set (*n* = 130)			
Mean age (range) (years)&	56 (18–80)	38.2 (20–75)	<0.0001
Sex (M:F)&	33∶30	40∶27	ns
Treatment Naïve: Treated*	28∶35	–	–
Gut: Pancreatic NENs	22∶3$	–	–
Independent Validation Set 1 (*n* = 115)			
Mean age (range) (years)&	50.4 (27–69)	38 (28–52)	<0.001
Sex (M:F)&	44∶28	26∶17	ns
Treatment Naïve: Treated*	16∶56	–	–
Gut: Pancreatic NENs	54∶18	–	–
Independent Validation Set 2 (*n* = 110)			
Mean age (range) (years)&	63.8 (40–83)	45.8 (24–75)	<0.0001
Sex (M:F)&	42∶29	25∶24	ns
Treatment Naïve: Treated*	9∶64	–	–
Gut:Pancreatic NENs	40∶25$$	–	–

* Treated includes surgical (hemicolectomy, ablation, liver resection) and chemotherapeutic/biological (sandostatin, temodar, RAD001, everolimus) therapies.

$ 3 patients were bronchopulmonary NENs.

$$ 6 patients categorized as “carcinoids of unknown primary”.

& Age−/sex-matching was not undertaken.

The majority >95% of patients were Caucasian.

**Table 2:** Clinical characteristics of patients (test and independent sets)**.**

Study Set	Primary Location									
**Test Set (** ***n*** ** = 63)** %	Lung	Stomach	Pancreas$	SI		Appendix		Colorectal		CUP
	3 (5%)	4 (6%)	3 (5%)	39 (62%)		8 (13%)		6 (9%)		0 (0%)
	**Grade** *									
	**G1**		**G2**				**G3**		**ND**	
	30 (48%)		18 (28%)				2 (3%)		13 (21%)	
	**Metastases** **									
	**No**				**Yes**			**ND**		
	12 (19%)				46 (73%)			5 (8%)		
**Study Set**	**Primary Location** &									
**Independent Validation Set 1 (** ***n*** ** = 72)** %	Lung	Stomach	Pancreas$	SI		Appendix		Colorectal		CUP
	0 (0%)	0 (0%)	18 (25%)	46 (64%)		3 (4%)		5 (7%)		0 (0%)
	**Grade** *									
	**G1**		**G2**				**G3**		**ND**	
	44 (61%)		20 (28%)				0 (0%)		8 (11%)	
	**Metastases** **									
	**No**				**Yes**			**ND**		
	10 (14%)				61 (85%)			1 (1%)		
**Study Set**	**Primary Location** &									
**Independent Validation Set 2 (** ***n*** ** = 71)** %	Lung	Stomach	Pancreas$	SI		Appendix		Colorectal		CUP
	0 (0%)	2 (3%)	25 (35%)	36 (51%)		0 (0%)		2 (3%)		6 (8%)
	**Grade** *									
	**G1**		**G2**				**G3**		**ND**	
	30 (42%)		9 (13%)				2 (4%)		30 (42%)	
	**Metastases** ** #									
	**No**				**Yes**			**ND**		
	1 (1%)				60 (85%)			10 (14%)		

CUP = carcinoid of unknown primary, ND = no data available, SI = small intestine.

* Grade: based on Ki67 or mitotic index (from WHO2010^29^).

** Metastases: any tumor disease identified in lymph nodes, mesentery, liver, lung, bone, ovary (or any combination thereof). Methodologies including octreoscan, identification at surgery, identification at pathology e.g. positive lymph nodes, etc.

& *p*<0.05 vs. Test set (Chi-square). This reflects the higher proportion (25–35%) of Pancreatic NENs included in the validation sets.

# *p*<0.05 vs. Test and Validation set 1 (Chi-square). This reflects the higher proportion ∼10% of patients with no metastases.

% A comparison of these clinical sets with the spectrum of disease included in the Surveillance Epidemiology and End Results (SEER) database for GEP-NENs^32,33^ identifies no significant differences. Patient characteristics also provide a reasonable reflection of the clinical spectrum of disease that is *pari passu* for NEN patients.

Details regarding Age and Sex for patients are included in Table 1.

$ Forty six of the samples were collected from pancreas, with the following break-down: ACTH (*n* = 1), gastrinoma (*n* = 2), glucagonoma (*n* = 1), insulinoma (*n* = 4), VIP (*n* = 3), Functional (no characterization of hormone, *n* = 8), non-functional (*n* = 27).

### Real-time PCR Analysis of Peripheral Blood Gene Expression

Real-time PCR was performed using Applied Biosystems products (details in **Supplemental Methods**). PCR values were normalized to *ALG9* (ΔΔC_T_) [15], using the control group as the population control (calibrator sample).

### Chromogranin A Measurement

CgA was measured using the DAKO ELISA kit (K0025, DAKO North America, Inc., Carpinteria, CA) [20] in a set of 176 samples (controls: *n* = 95; GEP-NENs: *n* = 81). A cut-off of 19 Units/L (DAKO) was used as the upper limit of normal.

### Classification Algorithms

Expression values were log-transformed and mapped to the range (1–100). GEP-NEN classifiers were built and optimized on the training set (*n* = 67 controls, *n* = 63 GEP-NENs) using 10-fold cross-validation design. In the internal training set, differentially expressed genes (control versus tumor) were calculated by a t-test. Four different learning algorithms [support vector machine (SVM), linear discrimination analysis (LDA), K-Nearest Neighbor (KNN), and Naive Bayes (Bayes)] were trained on the internal training set using the up-regulated features (uncorrected *p*<0.05). To control for over-fitting, the classifier was verified in 2 validation sets. A consensus labeling of “control” or “GEP-NEN” was generated by a “majority vote” approach [21], whereby a sample with <2 “control” predictions was designated as “GEP-NEN”. Detailed description of all classification algorithms is in **Supplementary Methods.**

All analyses were carried out using MATLAB’s Statistics and Bioinformatics toolboxes (2009a, The MathWorks, Natick, MA).

## Results

### Pipeline for Identifying and Defining Candidate Genes in GEP-NENs

#### 1. Gene co-expression network inference in GEP-NENs

We hypothesized that comparison of co-expression networks between GEP-NEN and other cancer datasets would provide additional biological insight. We utilized two independent GEP-NEN microarray datasets [15], [16] and compared them with well-characterized cancer datasets chosen for prevalence and represented by comprehensive microarray collections. Additionally, an independent normal human tissue dataset (79 different healthy tissues and cell types [2 replicates/tissue/cell type including liver, brain and heart, totaling 158 arrays] was included to eliminate co-expressions that may occur due to healthy tissue in malignant biopsies (**Table S1**).

**Figure 2:**
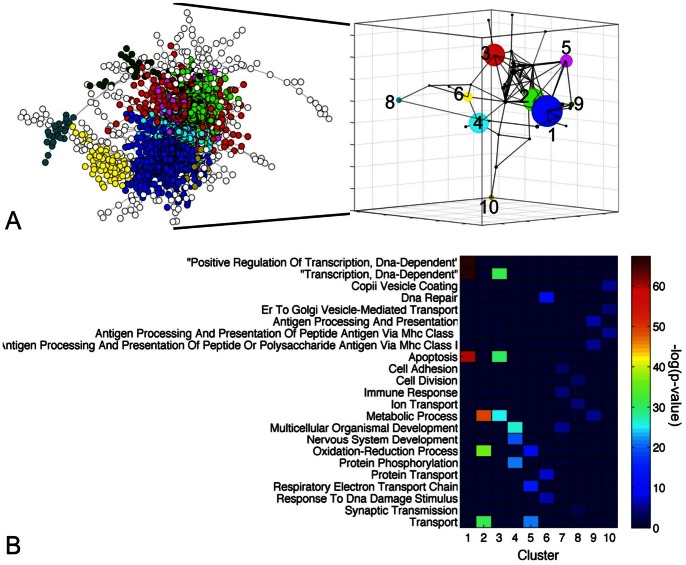
GEP-NEN gene co-expression network. **A)** Visualization of the GEP-NEN gene co-expression network (2545 genes, 30249 edges). Each node represents a gene, while a link represents a GEP-NEN-specific co-expression. Nodes that localized to the same network community are marked in the same color. The community structure of the GEP-NEN network is further visualized in the 3 dimensional inset, whereby each node represents a community while edges are drawn between communities that contain co-expressed genes. Larger nodes indicate bigger gene communities. **B)** Heatmap visualizing enrichment for over-represented Gene Ontology (GO) Biological Process (BP) terms assigned to the 10 largest clusters (>20 genes). Heatmap colors represent the significance of the enrichment.

Gene co-expression networks were constructed for all microarray datasets by linking genes whose expression correlated above a predefined PCC threshold (**Supplementary Methods**, **Figure S1**). Subsequently, the inference of a GEP-NEN network consisted of: 1) retaining co-expression pairs that recurred in both GEP-NEN datasets; 2) eliminating genes and co-expressions present in other cancer and normal tissue gene networks from the consensus GEP-NEN network; and 3) eliminating genes from the consensus GEP-NEN network that exhibited divergent changes in GEP-NEN-A and GEP-NEN-B datasets (**Figure 1**, **Figure S2**). This analysis produced 2892 genes and 30444 co-expressions. We focused on the largest connected component of this network (2545 genes and 30249 links), which contained 99% of all co-expressions (**Figure 2A**). It is important to note that a gene co-expression network does not attempt to identify “direct gene interactions” but rather contain “gene neighborhood relations” that are usually overlooked in conventional microarray analysis [22] and is used to identify genes that play distinct roles in a common pathway or biological process [23]. Therefore, functional characterization of a co-expression network should be regarded as a descriptive analysis aimed to generate additional testable hypotheses.

#### 2. Functional analysis of GEP-NEN gene co-expression network

To provide insight into molecular pathways captured by the GEP-NEN network, the DAVID functional enrichment tool was used to identify over-represented Biocarta, KEGG, and Reactome pathways (see **Supplemental** **Methods**). The most abundant pathways were Reactome pathways including “Integration of energy metabolism” (*n* = 58 genes, *p* = 4.2×10^−5^) and the “Diabetes pathway” (*n* = 68, *p* = 2.7×10^−4^), and KEGG pathways like “Pathways in cancer” (*n* = 72, *p* = 0.003) (**Table S2**). Other pathways included genes involved in immune responses, nervous system development, and metabolism. An important characteristic of most biological networks is that they tend to naturally organize into modules. We used the Louvain algorithm, a “greedy” method for iterative grouping of nodes into communities through modularity maximization [24], to partition the GEP-NEN network into 62 clusters with 800 and 3 genes in the largest and smallest clusters respectively (**Figure 2A**). Enrichment for over-represented GO-BP terms in clusters with >20 genes, revealed presence of processes including “Apoptosis” (*p* = 2.9×10^−26^, Cluster 1), “Oxidation reduction” (*p* = 2.3×10^−36^, Cluster 2), and “Nervous system development” (*p* = 7×10^−20^, Cluster 4) (**Figure 2B**). These processes are consistent with the known biology of GEP-NENs [25].

#### 3. Marker gene selection

We generated three panels of putative marker genes that were further examined by RT-PCR: 1) tissue-based panel, 2) peripheral blood-based panel and, 3) literature-curated panel. A detailed description of the methods is in **Supplementary Methods**.

To generate the ***tissue-based*** gene panel, we identified significantly (false discovery rate [FDR] adjusted *p*<0.025) up-regulated genes in both GEP-NEN-A and GEP-NEN-B datasets and retained only genes that were also present in the GEP-NEN gene co-expression network. Subsequently, we retained genes with high network clustering coefficient (≥0.25), based upon their increased likelihood of an association with tumorigenesis [26]. Finally, we examined a set of 369 genes that passed our filtering threshold using a manual literature-curated search. Our search criteria involved implication in: a) neuroendocrine axis, b) tumor formation, or c) metastasis. Using these constraints, 21 of the 369 “putative” marker genes were selected for PCR validation.

To derive a peripheral ***blood-based*** “putative” marker gene panel, we generated a transcriptome consisting of 14 peripheral blood samples (*n* = 7 controls, *n* = 7 GEP-NENs). There were 1382 significantly up-regulated (unadjusted *p*<0.05, FC>0) genes in GEP-NENs (details in **Supplemental Methods**). All genes with expression values in the lower 25^th^ quantile were excluded and only those genes with positive FC in both tissue datasets (GEP-NEN-A/B) were retained. This analysis produced 306 “putative” marker genes. A manual literature-curated search focusing on relevance to neuroendocrine biology/neoplasia identified 32/306 as putative targets for PCR validation.

The ***literature-curated*** *panel* consisted of 22 genes. Thirteen marker genes previously associated with GEP-NENs, either in our studies [11], [12] or in others [16], [27], were identified using queries of the Catalogue of Somatic Mutations in Cancer (COSMIC v60) database [28]. The additional 9 genes were included given their association with tumor initiation and metastasis.

Thus, based upon these analyses, 75 “putative” marker genes were selected for PCR analysis (**Figure 1** **, Figure S2**).

### Validation of GEP-NEN Marker Gene Panel in Test Set and Independent Sets

To validate a “putative” marker panel, transcript levels of mRNA isolated from a subset of the training set (controls: *n* = 49 and GEP-NENs: *n* = 28) was measured. This identified that 51 of the 75 candidate markers produced detectable product (C_T_<40 cycles) in blood. The 51 gene panel is listed (**Table 3** **, Table S3**).

**Table 3:** List of 51 marker genes.

ID	Gene Name
**AKAP8L**	A kinase (PRKA) anchor protein 8-like
**APLP2**	amyloid beta (A4) precursor-like protein 2
**ARAF**	v-raf murine sarcoma 3611 viral oncogene homolog
**ARHGEF40**	Rho guanine nucleotide exchange factor (GEF) 40
**ATP6V1H**	ATPase, H+ transporting, lysosomal 50/57kDa, V1 subunit H
**BNIP3L**	BCL2/adenovirus E1B 19kDa interacting protein 3-like
**BRAF**	v-raf murine sarcoma viral oncogene homolog B1
**C21orf7**	chromosome 21 open reading frame 7
**CD59**	CD59 molecule, complement regulatory protein
**COMMD9**	COMM domain containing 9
**CTGF**	connective tissue growth factor
**ENPP4**	ectonucleotide pyrophosphatase/phosphodiesterase 4 (putative function)
**FAM131A**	family with sequence similarity 131, member A
**FZD7**	frizzled homolog 7 (Drosophila)
**GLT8D1**	glycosyltransferase 8 domain containing 1
**HDAC9**	histone deacetylase 9
**HSF2**	heat shock transcription factor 2
**KRAS**	v-Ki-ras2 Kirsten rat sarcoma viral oncogene homolog
**LEO1**	Replicative senescence down-regulated leo1-like protein
**MKI67**	antigen identified by monoclonal antibody Ki-67
**MORF4L2**	mortality factor 4 like 2
**NAP1L1**	nucleosome assembly protein 1-like 1
**NOL3**	nucleolar protein 3 (apoptosis repressor with CARD domain)
**NUDT3**	nudix (nucleoside diphosphate linked moiety X)-type motif 3
**OAZ2**	ornithine decarboxylase antizyme 2
**PANK2**	pantothenate kinase 2
**PHF21A**	PHD finger protein 21A
**PKD1**	polycystic kidney disease 1 (autosomal dominant)
**PLD3**	phospholipase D family, member 3
**PNMA2**	paraneoplastic antigen MA2
**PQBP1**	polyglutamine binding protein 1
**RAF1**	v-raf-1 murine leukemia viral oncogene homolog 1
**RNF41**	ring finger protein 41
**RSF1**	remodeling and spacing factor 1
**RTN2**	reticulon 2
**SLC18A1**	solute carrier family 18 (vesicular monoamine), member 1
**SLC18A2**	solute carrier family 18 (vesicular monoamine), member 2
**SMARCD3**	SWI/SNF related, matrix associated, actin dependent regulator of chromatin, subfamily d, member 3
**SPATA7**	spermatogenesis associated 7
**SSTR1**	somatostatin receptor 1
**SSTR3**	somatostatin receptor 3
**SSTR4**	somatostatin receptor 4
**SSTR5**	somatostatin receptor 5
**TECPR2**	tectonin beta-propeller repeat containing 2
**TPH1**	tryptophan hydroxylase 1
**TRMT112**	tRNA methyltransferase 11-2 homolog (S. cerevisiae); similar to CG12975
**VPS13C**	vacuolar protein sorting 13 homolog C (S. cerevisiae)
**WDFY3**	WD repeat and FYVE domain containing 3
**ZFHX3**	zinc finger homeobox 3; hypothetical LOC100132068
**ZXDC**	ZXD family zinc finger C
**ZZZ3**	zinc finger, ZZ-type containing 3

#### 1. Utility of the 51 marker panel to identify GEP-NENs

**Figure 3:**
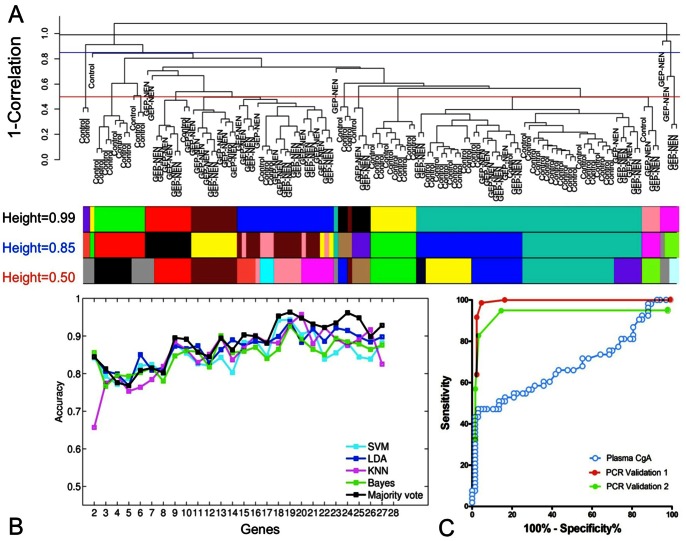
Utility of the 51 marker gene signature for identification of GEP-NEN disease. **A)** Unsupervised hierarchical clustering of the 130 samples in the training set (*n* = 67 controls, *n* = 63 GEP-NENs). Tree was generated with an average agglomeration method and 1-(sample correlation) was used as a measure of dissimilarity. Unique colors under the dendrogram represent sample cluster assignments, computed by cutting the hierarchical tree at height = 0.99 (black line), 0.85 (blue line), or 0.50 (red line) using a dynamic tree cutting approach [77]. **B)** Prediction accuracy of each classifier using sequential addition of 27 significantly up-regulated genes (*p*<0.05) in the GEP-NEN samples obtained using results of the 10-fold cross validation. **C)** Receiver operating characteristic (ROC) curves for “majority vote” classifier applied to validation sets 1 (AUC = 0.98, *p*<0.0001) and 2 (AUC = 0.95, *p*<0.0001) compared to ROC curve for utility of the plasma CgA values (AUC = 0.64, *p*<0.002). Direct comparisons of AUCs between set 1 or set 2 and CgA identified estimated Z-scores of 10.57 and 11.42 respectively, confirming the significant differences between the two detection systems (calculations detailed in **Supplementary Methods**).

The GEP-NEN classifiers were built on a training set (controls: *n* = 67, GEP-NENs: *n* = 63) and significantly up-regulated features between control and tumor cases were calculated by t-test (*n* = 27, *p*<0.05, **Figure 3A**). Four classification algorithms (SVM, LDA, KNN, and Bayes) and a 10-fold cross-validation design were used to build a classifier for the diagnosis of GEP-NENs. The average accuracy of the SVM, LDA, KNN, and Bayes algorithms to distinguish GEP-NEN from control samples using 27 genes was comparable –0.89 (0.85–1.0), 0.89 (0.86–0.93), 0.88 (0.85–0.93), and 0.86 (0.85–0.93) respectively. The “majority voting” combination of the four classifiers achieved an accuracy of 0.88 (**Figure 3B**). To control for over-fitting and to evaluate classifier performance, we examined two validation sets (see **Methods**). The “majority vote” classification was used to generate final predictions. In these validation sets, the performance metrics for differentiating GEP-NENs from controls exhibited sensitivities of 85–98% with specificities of 93–97%, PPVs of 95–96% and NPVs of 87–98%. The AUC for the diagnostic test in first and second validation sets were 0.98 and 0.95 respectively (**Figure 3C**). These results indicate the signature was effective at distinguishing between GEP-NENs and controls.

#### 2. Comparison of the 51 marker panel with Chromogranin A for GEP-NEN identification

**Figure 4:**
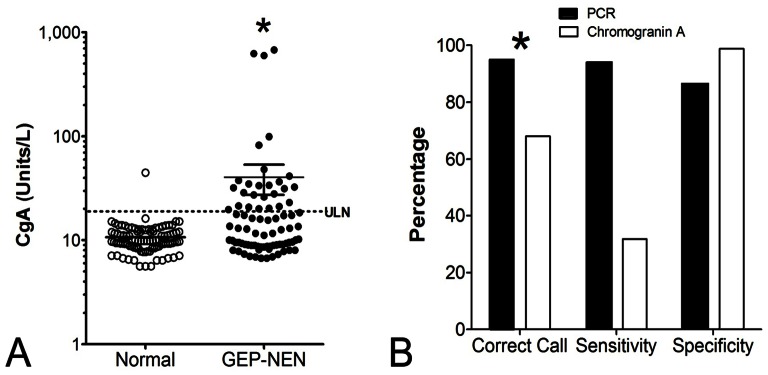
Comparison of the 51 marker gene signature with Chromogranin A (CgA) for detecting GEP-NENs. **A)** CgA levels were significantly elevated in the GEP-NEN group (*n* = 176; *p<0.002) but an overlap with normal values was identified. **B)** Comparison of the PCR-based approach with CgA protein measurement identified that call rates were significantly higher for the PCR-based test (*p<0.0005, χ^2^ = 12.3). The PCR blood test was significantly more accurate than measurement of CgA levels to detect GEP-NENs. ULN = upper limit of normal (19U/L – DAKO).

To examine the utility of the peripheral blood PCR signature, we compared it to measurements of CgA in a set (*n* = 176 samples). Levels of CgA were elevated (*p*<0.002) in GEP-NENs compared to controls (**Figure 4A**). Using the DAKO cut-off of 19 Units/L as the ULN, a total of 26 (32%) of 81 GEP-NENs were positive compared to 1 (1.0%) of 94 controls for performance metrics of 32% (sensitivity), 99% (specificity), 96% (PPV) and 63% (NPV). The correct call rate was 68%. A direct comparison of the molecular test and CgA ELISA identified that the PCR-based method had a significantly more accurate call rate compared to CgA levels (χ^2^ = 12.3, *p*<0.0005) (**Figure 4B**). The specificities were similar for detecting a GEP-NEN (94% versus 99%) but the sensitivity of the PCR test was significantly higher than for CgA (85% versus 32%).

### Additional Utility of GEP-NEN Marker Gene Panel

To further evaluate the potential utility of this marker panel, we undertook a sub-analysis of the data to examine whether there were any differences in sensitivity or specificity for detecting P-NENs versus GI-NENs and whether non-metastatic tumors could be detected. In addition, we wanted to determine how well the test performed in the patients with low CgA expression. We examined each of the validation sets (independent set 1 and 2) individually as well as the combination of the two sets.

**Figure 5:**
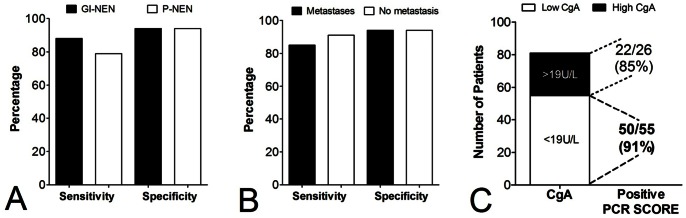
Utility of the 51 marker gene signature for detecting P-NENs, metastases and in patients with low Chromogranin A (CgA). **A)** The sensitivity and specificity of the test to detect GI-NENs (90%, 94%) and P-NENs (80%, 94%) was similar. **B)** The PCR-based approach could detect patients with no metastases as well as patients with metastases. **C)** The PCR-based test could accurately identify GEP-NENs even when plasma CgA were low (<19U/L). Overall, the PCR blood test was significantly more accurate than measurement of CgA levels to detect GEP-NENs (*p<10^−13^, χ^2^>50).

The performance metrics for identifying P-NENs were: sensitivity 64–100% and specificity 92–95%; overall 79% of the 43 pancreas NETs (in both sets) were positive by the test (specificity: 94%) (**Figure 5A**). For GI-NENs, this was 74–98% and 92–95%, respectively. Overall, 88% of the 95 GI-NENs (both sets) were positive (specificity: 94%). There was no significant difference (Chi-square = 1, *p* = 0.31, 2-tailed) indicating that the PCR test could identify these two tumor types with a similar efficacy.

Assessment of tumors with metastases identified an overall sensitivity and specificity of 85% (specificity: 94%) while 91% of the 11 documented patients with no metastases were positive (specificity: 94%). The PCR test therefore identified patients equally well irrespective of metastases (**Figure 5B**).

Using the 176 sample dataset for CgA and the DAKO cut-off of 19U/L, 55 patients were identified with low circulating levels of CgA. The PCR score in these patients was >2 in 50 (91%). For the 26 patients with elevated CgA, the PCR score was elevated in 22 (85%). Using diagnosis of GEP-NEN as a “standard”, the PCR score significantly outperformed measurements of CgA (Chi-square: >50, *p*<10^−13^) for the identification of the disease (**Figure 5C**).

## Discussion

We have developed and validated a PCR-based, blood-derived, molecular signature test that is based on 51 genes and identifies GEP-NENs with a high specificity and sensitivity. This test significantly outperforms the current CgA blood test that is used to confirm the clinical suspicion of a NEN. Since the blood PCR signature comprises 51 NEN-based transcripts that cover a wide biological spectrum, it is also more effective than a single peptide-based ELISA that identifies a secretory peptide unrelated to tumor cell proliferation and not produced by ∼25% of NENs [29], [30]. Such a multi-transcript approach is generally more effective than single parameter analyses [31], [32].

A key limitation of CgA measurement is that it only measures one variable of NENs namely a secretory peptide and the ELISA technique used is based on a number of different antibodies used by various commercial laboratories (e.g. Cisbio, DAKO or Eurodiagnostica). Measurements are thus not only mono-dimensional but not readily comparable if different assays utilizing different antibodies are used [29], [30], [33]. To ensure a broader biological coverage and diminish reliance on a single variable, we developed a multiple parameter PCR test.

A compendium of tissue-based and peripheral blood transcriptomes was used to develop a signature which exhibited GEP-NEN specificity and was biologically related to GEP-NENs. To generate a rational basis for integrating multiple transcripts a series of mathematical algorithms were utilized to derive the marker signature, namely the GEP-NEN classifier. These included gene co-expression network profiling and functional gene community detection, all robust methods previously used in the development of gene-based molecular protocols [34]. Experimental artifact was minimized and robustness amplified through the use of two independent GEP-NEN microarray datasets and seven normal and neoplastic tissue transcriptomes (total 551 arrays). To further assure the biological relevance of the analysis functional enrichment of genes associated with GEP-NENs (inclusion of GO-BP terms such as “Chromatin organization”, “Negative regulation of gene expression”, and “Cell surface receptor linked signal transduction” [25] [e.g., chromogranin A/B (CHGA/CHGB: secretion], glutamate decarboxylase 1 [GAD1: GABA production] [25] and Aurora kinase B [AURKB: mitosis] [35] was undertaken.

Since a key component of accuracy was dependent on accurate and reproducible mathematical analysis we utilized supervised learning methods, SVM, LDA, KNN, and Bayes to build the GEP-NEN classifier. These strategies have previously been used as broad applications in two-class classification problems in biomedicine. SVM has been utilized to predict grading in astrocytomas [36] (>90% accuracy), and prostatic carcinomas (74–80% accuracy) [37]. LDA can detect non-small cell lung cancer in peripheral blood [38], while KNN models have been used to predict outcome in neuroblastoma [39]. The Bayes classifier has been used to predict prostate cancer recurrence [40]. Each therefore has utility for identifying individual or multi-variable alterations in neoplasia. Combining these techniques with a “majority vote” strategy in two independent validation cohorts, the PCR-based test exhibited correct call rates of 91–97% with sensitivities and specificities of 85–98% and 93–97% respectively for the identification of GEP-NENs. These performance metrics are comparable to similar algorithms that were successfully used clinically to detect CTCs e.g. cutaneous T-cell lymphoma (90%) [41].

To assess the efficacy of this signature index, we then compared it to CgA which is the current NEN marker used to establish diagnosis and disease status [30], [42]–[46]. CgA elevations are considered a sensitive, ∼60–85% accurate marker for GEP-NENs [1]. Measurements are, however, non-specific (10–35%) since CgA is elevated in a wide variety of diverse conditions [30], [33], [43]. These include non endocrine neoplasia (pancreatic and prostate) and a wide variety of cardiac, endocrine and inflammatory diseases [47], as well as in patients undergoing acid suppressive therapy with the proton pump inhibitor (PPI) class of drugs [48] and in renal failure [49]. CgA is constitutive component of neuroendocrine secretion, not proliferation, and therefore its use as a surrogate marker for tumor growth has obvious limitations [1]. In the current study we compared the PCR test with a widely available commercial CgA kit (DAKO: K0025) [30], [33]. Values were, as expected, elevated in GEP-NENs but exhibited a significant overlap with controls with an accuracy of 60% and sensitivity of 32%. It is likely that use of other kits to measure CgA would generate similar numbers given their published concordance (∼40–70%) [30], [33]. In comparison, the PCR-based test exhibited a sensitivity of >85% with a correct call rate of >90%. Evaluation of the ROCs was similarly significantly effective for the PCR-based test, which exhibited an AUC of 0.95–0.98 compared to 0.64 for the CgA. AUCs for CgA have ranged as high as 0.8–0.9 in other studies [50], [51], but this is dependent both on the kits used, the patient inclusion criteria e.g., undergoing treatment or type of GEP-NEN, but most importantly, the cut-off chosen, which is often population-dependent [30]. In comparison to other molecular-based tests, the performance metrics for the NEN-PCR-based test are substantially higher than for prostate (PSA or PMSA (0.75, both single target test) [52] or colon cancer (0.51–0.72, a two target PCR test) [53]. Given the utility of these latter cancer tests in clinical management [54], it is probable that application of this PCR multi-transcript measurement strategy to GEP-NENs will be similarly effective.

It has been noted that the majority of biomarker studies may not translate into clinically relevant tests [55]. For example, peripheral blood screens for colorectal cancer are not routine practice [56]. This is paradoxically associated with the sensitivity of PCR *per se*. Substantial differences in final yield can occur if there are minor variations in reaction components and thermal cycling conditions and/or mispriming events during PCR [57]–[61]. To minimize these potential issues, we have chosen to use a TAQMAN approach. In other studies, this has been demonstrated to have a low variability between runs ranging between 0–5% [62], have small coefficient of variations (CVs) for the cycling threshold (C_T_) of 1–3% [63] and results in acceptable CVs for normalized data between 10–24% [64], [65]. A consistent protocol for RNA isolation, cDNA synthesis and real-time PCR is considered appropriate to provide a stable platform for target and housekeeping gene analyses [57], [62], [63], [66], [67]. Stringent quality control [68], standardization of sample acquisition [69] and processing [70] therefore are a prerequisite for use of this molecular tool which makes it likely that any PCR approach will require dedicated, specialized facilities.

Irrespective of the potential limitations, our study identifies that a PCR-based test is significantly more sensitive than that currently utilized, namely CgA measurements, and can detect the majority (∼95%) of patients with disease irrespective of the location, extent, grade or metastasis. It is therefore likely that the test would be useful in a number of areas, following appropriate study. One is as a “rule-out” diagnostic test (to confirm absence of a GEP-NEN or residual disease). The low incidence of GEP-NENs in the population makes it unlikely to be cost-effective as a screening tool for tumor detection. The high sensitivity of the PCR test, in contrast, renders it a more effective tool to rule out a diagnosis. This will eliminate the relatively large number of “borderline” abnormal CgA results, particularly when different types of kits are used. Any future studies, should, in addition, assess whether medications or conditions associated with non-specific elevations in neuroendocrine cell numbers, e.g., PPIs, increase transcript expression. Given the similarities in biology (i.e., expression of receptors, pathways involved in secretion, molecular pathways e.g., MEN-I) [71]–[73] between GEP-NENs and other NENs e.g., pheochromytomas or medullary thyroid cancers, it would be useful to assess whether the PCR test can accurately identify these lesions. The existence of tumors with a significant neuroendocrine component e.g., prostate tumors [74] or colorectal cancers [75], [76], provides additional clinical samples in which to evaluate the efficacy of the PCR test.

Currently, CgA is used to evaluate treatment protocols [4], [46] as expression levels are considered to relate to tumor burden [46]. However, issues remain with the use of different measurement protocols as well as how to accurately assess CgA in monitoring disease if values are low or within the normal range. Given the high rate of detection even when plasma CgA levels are low (91% of these samples could be accurately identified by the PCR test), we anticipate that the PCR test can potentially be used as a prognostic. Future studies examining whether the PCR test results alter in response to therapy e.g., debulking or targeted therapy, would answer this possible indication.

In conclusion, using computational and machine learning approaches, including analysis and integration of tumor tissue and circulating peripheral blood transcripts, we identified a panel of 51 marker genes selectively associated with GEP-NENs. The test can differentiate between GEP-NENs and controls and has a high PPV and NPV (>90%). It is more accurate than the currently used clinical standard CgA assay, which identifies a single peptide related only to tumor secretion. The PCR-based signature measures multiple transcripts which reflect the diverse biological profile of a proliferating NEN and may, with further examination in appropriate studies, be tested as a measure of tumor responsiveness and, potentially, as a prognostic.

## Supporting Information

Figure S1 **Selection of Pearson correlation coefficient thresholds for gene co-expression network inference.**
(TIF)

Figure S2 **Computational pipeline used to derive a set of 51 markers that identify GEP-NEN disease.**
(TIF)

Table S1 **Microarray Datasets used in GEP-NEN network analysis.**
(DOCX)

Table S2 **Functional enrichment of 2545 genes in the GEP-NEN network for Biocarta, KEGG, and Reactome pathways.**
(DOCX)

Table S3 **List of 51 marker genes used in the study.**
(XLS)

Methods S1 (DOCX)
