# Supplementary figures and images for: The Identification of Gut Neuroendocrine Tumor Disease by Multiple Synchronous Transcript Analysis in Blood
Source: PLoS One. 2013 May 15;8(5):e63364. doi: 10.1371/journal.pone.0063364 (PMC3655166; doi:10.1371/journal.pone.0063364)

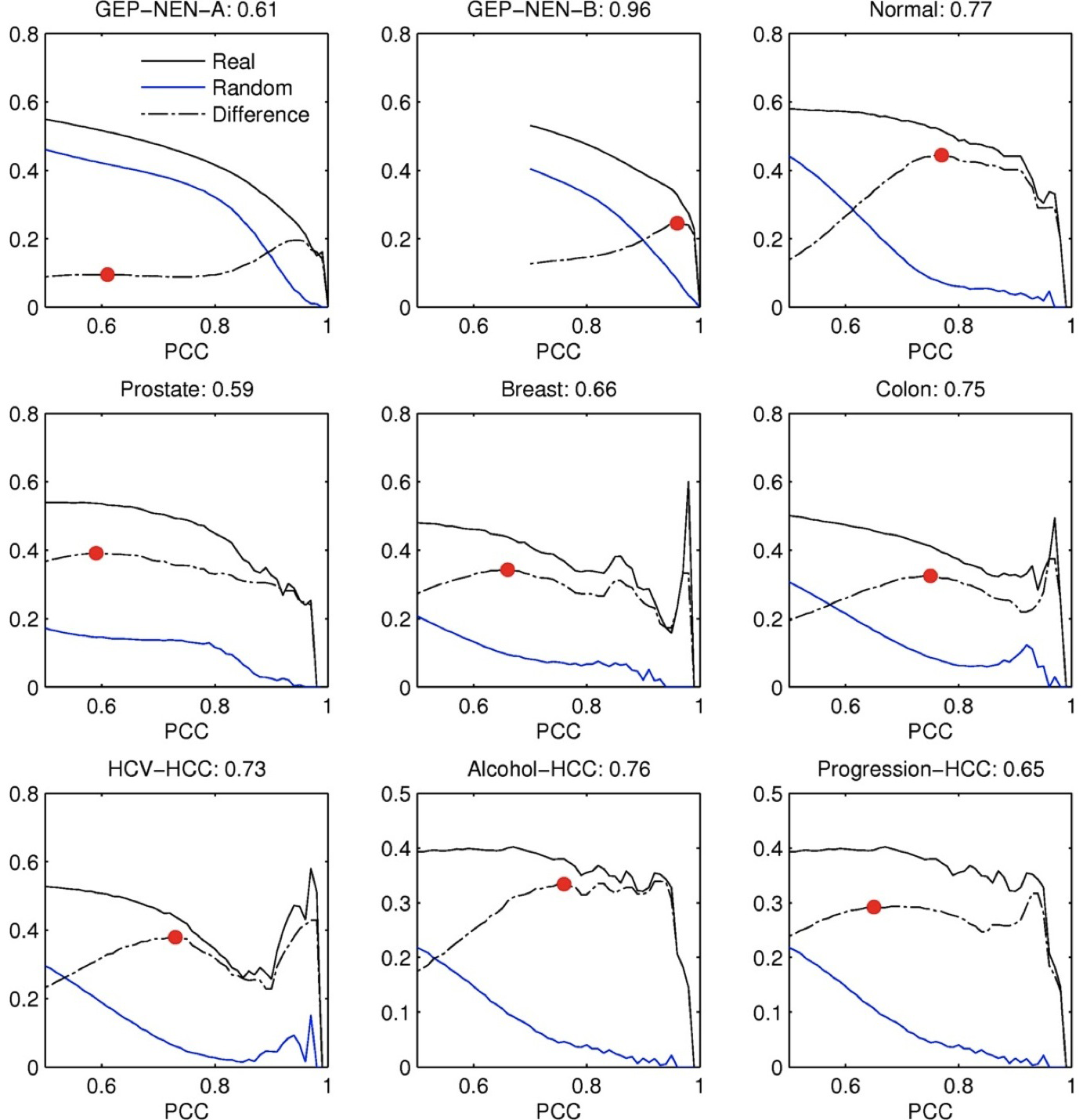

Supplement: Figure S1 — Selection of Pearson correlation coefficient thresholds for gene co-expression network inference. (TIF) [file pone.0063364.s001.tif]

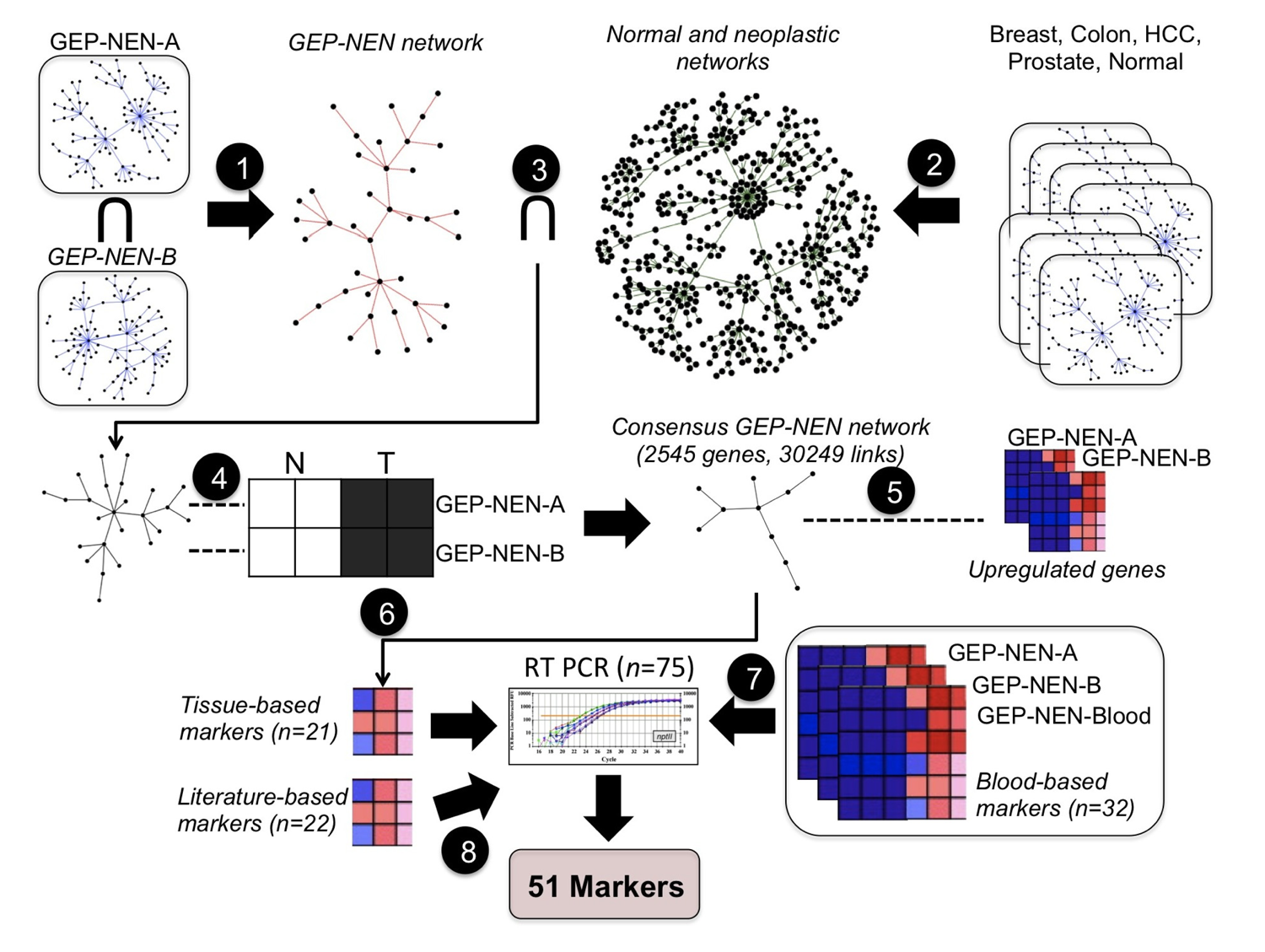

Supplement: Figure S2 — Computational pipeline used to derive a set of 51 markers that identify GEP-NEN disease. (TIF) [file pone.0063364.s002.tif]
