# Supplementary material for: The Identification of Gut Neuroendocrine Tumor Disease by Multiple Synchronous Transcript Analysis in Blood
Source: PLoS One. 2013 May 15;8(5):e63364. doi: 10.1371/journal.pone.0063364 (PMC3655166; doi:10.1371/journal.pone.0063364)
Supplement: Table S1 — Microarray Datasets used in GEP-NEN network analysis. (DOCX) [file pone.0063364.s003.docx]

**Supplemental Table A.** Microarray Datasets used in GEP-NEN network analysis

| **Dataset** | **Conditions** | **Platform** | **PCC cutoff** | **Accession Number** |
| --- | --- | --- | --- | --- |
| Alcohol-HCC  Dataset | Normal Liver, HBV/HCV/Alcoholic Cirrhosis, HCC (*n*=65) | HG-U133A | 0.76 | E-TABM-36 |
| Viral-HCC  Dataset | Normal Liver, HCV-HCC (*n*=124) | HG-U133A_2 | 0.73 | E-GEOD-14323 |
| Progression-HCC Dataset | Normal Liver, HCV-Cirrhosis, 4 stages of HCV-HCC (*n*=75) | HG-U133A Plus 2 | 0.65 | E-GEOD-6764 |
| Breast  Dataset | Normal, breast tumor (*n*=86) | HG-U133A | 0.66 | E-GEOD-15852 |
| Colon  Dataset | Normal, colon cancer (*n*=47) | HG-U133A | 0.75 | E-MTAB-57 |
| Prostate  Dataset | Normal, prostate cancer (*n*=154) | HG-U133 Plus 2 | 0.59 | E-GEOD-17951 |
| Normal Set | Normal tissues and cell lines (*n*=158) | HG-U133A | 0.77 | E-TABM-145 |
| GEP-  NEN-A | Normal tissue, primary NEN, metastatic NEN (*n*=13) | HG-U133A | 0.61 | E-GEOD-6272 |
| GEP-  NEN-B | Normal tissue, primary NEN, metastatic NEN (*n*=12) | HG-U133 Plus 2 | 0.96 | E-TABM-389 |

HCC=hepatocellular carcinoma; HBV=hepatitis B virus; HCV=hepatitis C virus; NEN=neuroendocrine neoplasm
