# Supplementary material for: The Identification of Gut Neuroendocrine Tumor Disease by Multiple Synchronous Transcript Analysis in Blood
Source: PLoS One. 2013 May 15;8(5):e63364. doi: 10.1371/journal.pone.0063364 (PMC3655166; doi:10.1371/journal.pone.0063364)
Supplement: Table S2 — Functional enrichment of 2545 genes in the GEP-NEN network for Biocarta, KEGG, and Reactome pathways. (DOCX) [file pone.0063364.s004.docx]

**Supplementary Table B.** Functional enrichment of 2545 genes in the GEP-NEN network for Biocarta, KEGG, and Reactome pathways.

| **Category** | **Term** | **Count** | **P-Value** | **Bonferroni** |
| --- | --- | --- | --- | --- |
| Reactome | REACT_1505:Integration of energy metabolism | 58 | 4.17E-05 | 0.0027 |
| KEGG | hsa00020:Citrate cycle (TCA cycle) | 15 | 1.27E-04 | 0.0240 |
| Reactome | REACT_15380:Diabetes pathways | 68 | 2.66E-04 | 0.0171 |
| Reactome | REACT_474:Metabolism of carbohydrates | 29 | 4.09E-04 | 0.0263 |
| Reactome | REACT_602:Metabolism of lipids and lipoproteins | 38 | 0.0028 | 0.1646 |
| KEGG | hsa00330:Arginine and proline metabolism | 18 | 0.0029 | 0.4239 |
| KEGG | hsa05200:Pathways in cancer | 72 | 0.0039 | 0.5281 |
| KEGG | hsa00051:Fructose and mannose metabolism | 13 | 0.0049 | 0.6115 |
| Reactome | REACT_13685:Synaptic Transmission | 23 | 0.0059 | 0.3199 |
| Reactome | REACT_1046:Pyruvate metabolism and TCA cycle | 11 | 0.0060 | 0.3222 |
| KEGG | hsa00071:Fatty acid metabolism | 14 | 0.0077 | 0.7724 |
| KEGG | hsa04810:Regulation of actin cytoskeleton | 49 | 0.0092 | 0.8284 |
| KEGG | hsa04730:Long-term depression | 20 | 0.0107 | 0.8721 |
| KEGG | hsa03320:PPAR signaling pathway | 20 | 0.0107 | 0.8721 |
| Reactome | REACT_13698:Regulation of beta-cell development | 10 | 0.0110 | 0.5143 |
| KEGG | hsa00010:Glycolysis / Gluconeogenesis | 18 | 0.0115 | 0.8892 |
| KEGG | hsa04530:Tight junction | 33 | 0.0117 | 0.8943 |
| KEGG | hsa04360:Axon guidance | 32 | 0.0118 | 0.8971 |
| KEGG | hsa05216:Thyroid cancer | 11 | 0.0120 | 0.8996 |
| KEGG | hsa04614:Renin-angiotensin system | 8 | 0.0121 | 0.9019 |
| KEGG | hsa00601:Glycosphingolipid biosynthesis | 10 | 0.0124 | 0.9085 |
| KEGG | hsa04520:Adherens junction | 21 | 0.0173 | 0.9643 |
| KEGG | hsa04720:Long-term potentiation | 19 | 0.0192 | 0.9755 |
| Reactome | REACT_13:Metabolism of amino acids | 37 | 0.0195 | 0.7221 |
| Reactome | REACT_1698:Metablism of nucleotides | 20 | 0.0279 | 0.8416 |
| KEGG | hsa05219:Bladder cancer | 13 | 0.0291 | 0.9965 |
| KEGG | hsa00650:Butanoate metabolism | 11 | 0.0369 | 0.9992 |
| KEGG | hsa04950:Maturity onset diabetes of the young | 9 | 0.0370 | 0.9993 |
| BIOCARTA | h_CSKPathway:Activation of Csk by cAMP-dependent Protein Kinase Inhibits Signaling through the T Cell Receptor | 8 | 0.0371 | 1.0000 |
| KEGG | hsa00561:Glycerolipid metabolism | 13 | 0.0480 | 0.9999 |
| KEGG | hsa05211:Renal cell carcinoma | 18 | 0.0489 | 0.9999 |
| Reactome | REACT_11123:Membrane Trafficking | 12 | 0.0489 | 0.9616 |

The entire human genome was used as reference and *p*<0.05 was considered significant.
