# Supplementary material for: The Identification of Gut Neuroendocrine Tumor Disease by Multiple Synchronous Transcript Analysis in Blood
Source: PLoS One. 2013 May 15;8(5):e63364. doi: 10.1371/journal.pone.0063364 (PMC3655166; doi:10.1371/journal.pone.0063364)
Supplement: Methods S1 — (DOCX) [file pone.0063364.s006.docx]

**Supplementary Methods**

**Microarray acquisition and pre-processing**

Raw and processed gene expression datasets were obtained from the ArrayExpress database [[1](#_ENREF_1)]. Raw expression values were normalized using Robust Multi-array Average (RMA) [[2](#_ENREF_2)] pipeline available through the *affy* [[3](#_ENREF_3)] package for R statistical environment. RMA normalization consists of three steps: a background adjustment, quantile normalization of log_2_ intensity values, and summarization. Previous comparisons of RMA to other microarray normalization approaches such as MAS 5.0 [[4](#_ENREF_4)] and dChip [[5](#_ENREF_5)] have demonstrated that RMA 1) is more precise, particularly for low expression values, 2) provides more consistent estimates of fold change, and 3) provides higher specificity and sensitivity to detect differential expression [[6](#_ENREF_6)].

For arrays where raw gene expression values were not available, signal intensities were log_2_-transformed. In all cases where multiple probesets mapped to a single gene, only median signal intensity was retained. It is possible that multiple probe sets assigned to the same gene could capture alternative splicing, use of alternative poly(A) sites, or errors associated with an experimental protocol [[7](#_ENREF_7)]. Given that it is difficult to distinguish the biological phenomenon from technical error, we considered the median intensity value of probesets that mapped to the same gene to simplify integration of co-expression networks across multiple microarray datasets. This was in accordance to other studies, where multiple probe intensities were merged in a similar fashion [[8](#_ENREF_8)].

To standardize annotation across microarray platforms, Affymetrix probe identifiers were mapped to their corresponding Ensembl (accessed March 23, 2011) gene identifiers (IDs) [[9](#_ENREF_9)].

**Graph theoretic analysis**

*GEP-NEN co-expression network reconstruction*

Pairwise similarity in gene expression vectors was expressed by the Pearson correlation coefficient (PCC). Gene pairs that correlated above a predefined PCC threshold were represented in the form of an undirected unweighted network, where nodes correspond to genes and links (edges) correspond to co-expression between genes. A data-driven computational method was used to calculate the appropriate PCC threshold for each microarray dataset [[10](#_ENREF_10)]. This procedure involves comparing the observed network clustering coefficient and its randomized counterpart as the number of connections is gradually decreased. This is achieved by increasing edge weight threshold. In the case of gene co-expression networks, edge threshold is systematically tested for absolute PCC values between 0 and 1. Genes and co-expressions above a specific PCC threshold are then represented as nodes and edges in an undirected and unweighted network and average real clustering coefficient is computed. Subsequently, a randomized network is generated by rewiring edges in the original co-expression network while preserving the degrees of the respective nodes, as described by Maslov & Sneppen [[11](#_ENREF_11)]. The average clustering coefficient of this random network is then calculated. Finally, the cut-off threshold *C** is determined by incrementally testing all edge thresholds and finding the threshold which corresponds to the first local maximum of the observed difference curve *C^Clu^* − *C_0_^Clu^*. Here, *C^Clu^* and *C_0_^Clu^* are average clustering coefficients of the real and corresponding randomized networks with preserved degree distributions at each PCC threshold. Overall, this strategy has been previously published [[10](#_ENREF_10)] and applied to reverse-engineering gene co-expression networks in hepatocellular carcinoma [[12](#_ENREF_12)].

*Generation of random networks*

To create random networks as part of our PCC inference strategy, we used the Maslov-Sneppen approach [[11](#_ENREF_11)] (MATLAB code is available on the author’s website: <http://www.cmth.bnl.gov/~maslov/matlab.htm>). Randomization was performed by rewiring edges in the original networks while preserving degrees of the respective nodes. The number of rewiring steps taken for each model was 4X(number of edges) [[11](#_ENREF_11)].

*Computing clustering coefficient*

Clustering coefficient, *C^Clu^*, intends to answer the question: in what percentage of cases, a node’s neighbors are also neighbors. For node *i*, *C^Clu(i)^* is defined as:

$C^{Clu(i)}=\frac{2n}{k_{i}(k_{i}-1)}$,

where *n* denotes the number of direct links connecting the *k_i_* nearest neighbors of node *i*. *C^Clu^* ranges from zero (for a node that is part of a loosely connected group) to one (for a node at the center of a fully connected cluster). Thus, *C^Clu^* measures the local clustering in the graph. In protein-protein interaction networks, densely-clustered gene neighborhoods appear to be essential for cell survival [[13](#_ENREF_13)]. Additionally, high clustering coefficients were previously shown to be hallmarks of cancer-associated genes [[14](#_ENREF_14),[15](#_ENREF_15)].

*Co-expression network partitioning*

We used the Louvain algorithm to partition the GEP-NEN network. This method is a greedy optimization procedure that attempts to optimize the modularity of a partition of the network [[16](#_ENREF_16)]. Modularity, *Q*, is defined as the fraction of all edges that lie within communities minus the expected value of the same quantity in a graph in which the vertices have the same degrees but edges are placed randomly [[17](#_ENREF_17),[18](#_ENREF_18)]. It is given by:

$$Q=\sum_{s=1}^{NC} \frac{E_{s}}{E}-\left(\frac{k_{s}}{2E}\right)^{2}$$

Where *NC* is the number of clusters, *E* is the number of edges in the network, *E_s_* is the number of edges between vertices within cluster *s*, and *k_s_* is the sum of the degrees of the vertices in cluster *s*. The value of the modularity measure *Q* ranges from 0 to 1, and the optimal clustering is achieved by maximizing *Q*.

The Louvain approach consists of two phases. First, the algorithm searches for "small" communities by optimizing network modularity in a local way. Second, it aggregates nodes of the same community and builds a new network whose nodes are the communities. These steps are repeated iteratively until a maximum of modularity is attained.

Previously, we applied the Louvain algorithm to identify gene co-expression communities involved in secretory response of neuroendocrine tumors [[19](#_ENREF_19)], while other groups have used it to uncover hierarchical modularity in brain functional networks [[20](#_ENREF_20)] and describe community structure in human social networks [[21](#_ENREF_21)].

*Gene co-expression network enrichment*

The enrichment analysis of gene co-expression clusters was performed using the hypergeometric test. For a cluster with *n* genes and an *a priori* defined functional category with *K* genes, the hypergeometric test is used to evaluate the significance of overlap *k* between the module and category [[22](#_ENREF_22),[23](#_ENREF_23)]. All *N* genes in a network were used as reference. The significance of overlap is then calculated as:

$$p=\sum_{i=k}^{n} \frac{\left(\begin{matrix} N-K \\ n-i \end{matrix}\right)\left(\begin{matrix} K \\ i \end{matrix}\right)}{\left(\begin{matrix} N \\ n \end{matrix}\right)}$$

To avoid too general or specific annotation terms, only Gene Ontology (GO) Biological Process (BP) categories with more than 5, but fewer than 1000 genes were retained.

*Functional enrichment analyses*

Clusters of genes in a co-expression network were identified [[24](#_ENREF_24)] and enriched for over-represented Gene Ontology (GO) Biological Process (BP) terms using the hypergeometric test. All genes in a network were used as a reference. We excluded terms with <5 or >1000 assigned genes. The Database for Annotation, Visualization and Integrated Discovery (DAVID) [[25](#_ENREF_25)] was used for statistical enrichment of gene sets for GO-Fat terms. This was also used to identify over-represented Biocarta (http://www.biocarta.com/genes/index.asp), Kyoto Encyclopedia of Genes and Genomes (KEGG), and Reactome pathways in the GEP-NEN co-expression network. Network inference, gene community detection, and topological analysis was carried out using the Functional Genomics Assistant (FUGA) toolbox [[23](#_ENREF_23)].

**Differential expression analysis**

All differential expression analyses, including fold change (FC) calculations and Benjamini and Hochberg’s False Discovery Rate (FDR) adjustments were performed using the Linear Models for Microarray Data (*limma*) [[26](#_ENREF_26)] package for R statistical environment. The aim of the *limma* package is to ﬁt a linear model to the expression data for each gene, making the analyses stable even for experiments with small number of arrays.

**Classification algorithms**

We used four different learning algorithms to derive discrimination rules. All algorithms are available as parts of the MATLAB’s Statistics and Bioinformatics toolboxes (2009a, The MathWorks, Natick, MA) and were used with default parameters.

*Support vector machine (SVM)*

MATLAB commands: *svmtrain* and *svmclassify* (Statistics toolbox) with default parameters. SVM is a non-probabilistic binary classifier that performs classification tasks by constructing hyperplanes in a multidimensional space and separating cases of different class labels. The algorithm takes a set of input data and predicts, for each given input, which of two possible classes (GEP-NEN or Control) forms the input. Previously, SVM has been utilized to predict grading in astrocytomas [[27](#_ENREF_27)] (>90% accuracy), and prostatic carcinomas (74-80% accuracy) [[28](#_ENREF_28)].

*Linear discrimination analysis (LDA)*

MATLAB commands: *classify* (Statistics toolbox) with the default 'linear' type parameter. LDA aims to find a linear combination of features that characterizes or separates two or more classes of objects or events. In our dataset, we aimed to separate GEP-NENs from Controls. The algorithm is similar to regression analysis, however unlike the latter, the dependent variable is a categorical quantity. Previously, LDA has been used to detect non-small-cell lung carcinoma (NSCLC) in peripheral blood [[29](#_ENREF_29)].

*K-Nearest Neighbor (KNN)*

MATLAB commands: *knnclassify* (Statistics toolbox) with default parameters. KNN algorithm is perhaps the most intuitive classifier among the multiple machine learning techniques. Classiﬁcation is achieved by identifying the nearest neighbors of example query in a training set and using those neighbors to determine the class of the query. Previously KNN models have been used to predict outcome in neuroblastoma [[22](#_ENREF_22)] (a tumor type considered as part of the NEN “group”).

*Na*ï*ve Bayes (Bayes)*

MATLAB commands: *NaiveBayes.fit* and *NaiveBayes.predict* (Statistics toolbox) with default parameters. Bayes classifier is a probabilistic classifier based on applying Bayes' theorem with strong (naïve) independence assumptions. The algorithm assumes that the conditional probabilities of the independent variables are statistically independent. Importantly, samples in the test set are labeled according to class that achieves the highest posterior probability after applying the Bayes' rule. Normal distribution fit was used to model data, while empirical prior probabilities were used to estimate relative frequencies of classes in the training dataset. Previously, Naïve Bayes classifier has been used to identify novel housekeeping genes [[30](#_ENREF_30)] and prostate cancer recurrence [[31](#_ENREF_31)].

Each classifier was built and optimized on the training set (*n*=67 controls, *n*=63 GEP-NENs) using 10-fold cross-validation design: the original sample is randomly partitioned into 10 subsamples and the classifier is trained on the 9 subsamples and tested on the 1 subsample. The cross-validation process is then repeated 10 times (the folds), with each of the subsamples used once.

The classifier performance was evaluated using MATLAB’s *classperf* class with default parameters, available from the Bioinformatics toolbox.

**Sample collection and analysis for blood-based PCR**

*Clinical Data*

Information informing the clinical characteristics are included in **Table 2**. Included are the location of the primary, the Grade (based on WHO2010 criteria) [[32](#_ENREF_32)], and evidence for any metastasis. The blood-based PCR test was developed and tested in patients who have small intestinal and pancreatic tumors (67-86% of patients), that are principally grade 1 and 2 lesions (Ki67<20%) (76-89%) and have metastases (73-85%). The blood-based PCR test was developed to identify patients that have GEP-NEN disease at a variety of different sites, and can identify both low proliferating and well as higher proliferating tumors. Both metastatic and non-metastatic samples were included in the cohorts. The majority of patients (>95%) were Caucasian. These patient characteristics provide a reasonable reflection of the clinical spectrum of disease that is *pari passu* for NEN patients. Furthermore, a comparison of these clinical sets with the spectrum of disease included in the Surveillance Epidemiology and End Results (SEER) database [[33](#_ENREF_33),[34](#_ENREF_34)] identifies no significant differences, thus confirming the utility of these datasets for the analyses that were undertaken.

*Inclusion and exclusion criteria for cases and controls*

Cases were prospectively collected at three institutions (Yale/Charité/Mayo) from patients attending out-patient clinics following ethics approval from each of the institutions. Inclusion criteria included the following: proven GEP-NEN or bronchopulmonary NEN (any stage, any grade) (no limitation on current or previous therapy, no limit on age or sex). Exclusion criteria included age (<18 years). All (207/207) were included in the final study. Fifty nine percent of NENs were collected at Yale University; 27% from the Mayo Clinic and 14% from Berlin.

Controls were prospectively collected at two institutions (Yale/Charité) both from GEP-NEN family members as well as non-family members e.g., care-givers, or friends and colleagues who attended the clinics. Inclusion criteria included: age 18 to 80 years, apparently healthy blood donors, with no previous history of neoplasia at the time of recruitment/blood collection. Exclusion criteria included: age (<18 years), evidence of neoplasia, history of IBD (Crohn’s or Ulcerative colitis), current use of proton pump inhibitors or H_2_ blockers. One hundred and fifty nine (96%) of 165 were included in the final study. Cases and controls were included on entry into the consecutive sets – test set, validation set 1 and validation set 2. No age-/sex-matching were undertaken.

*Methods of storage of samples*

All blood samples were collected in 9mg K_2_EDTA tubes (BD Vacutainer Venous Blood Collection Tubes, BD Diagnostics). Aliquots of whole blood were stored at -80°C within 2 hrs of collection (samples immediately stored on ice/4°C after sampling) per standard molecular diagnostics protocols [[35](#_ENREF_35)].

*Sample blinding with respect to case/control classification*

All samples were de-identified and recoded at each of the institutions e.g., YALE1-, MC1-, BER1- etc. Classifiers including case/control were not available prior to running the MATLAB classification algorithms. For data input, samples were encoded S1-S130 (e.g., for the training set, or S1-S115 for validation set 1 etc) prior to analysis. The code was broken to analyze the MATLAB output (a .csv file with values of “normal” or “tumor” for each algorithm for each sample. S-values were compared to pre-identified values at this stage.

*Time frame over which samples were collected*

Samples were collected between March 2008-2012.

*Sample size rationale, power calculations*

We undertook to approaches to identify sample size and determine the effect size. The first approach can be considered “heuristic”, the second “statistical”.

1. Heuristic approach: Sample size calculation identified that for this unmatched case-control study, using a bilateral unpaired Student's t test with protection against type I error of 5 and 80% of power, a total of 10 cases and 10 controls would be required [[36](#_ENREF_36),[37](#_ENREF_37)]. However, an analysis of data from modeling transcript variance in disease states [[38](#_ENREF_38),[39](#_ENREF_39)] identified that a sample sizes (N) should be at least > (J + 3)/2, where J is the number of genes examined. Based on a 51 gene panel, the sample size was calculated to be at least >27. We therefore included at minimum of 27 samples for each analysis undertaken. For the Test set, the power was 1 (alpha = 0.05). For the Validation set 1 and 2, the power was 1. This approach provides the “lower” limit of samples required for analysis.
2. Statistical approach: Sample size and power calculations were performed using the *pwr* package for R statistical environment. The package implements Cohen’s [[40](#_ENREF_40)] power analysis functions. First, we determined the necessary number of cases to detect GEP-NENs with sensitivity of 0.95 and power of 0.95, providing that we considered acceptable sensitivity to be 0.80. This sensitivity limit has previously been applied in biomarker evaluations in Alzheimer disease [[41](#_ENREF_41)], inflammatory arthritis [[42](#_ENREF_42)], and bladder cancer [[43](#_ENREF_43)]. The significance level was controlled at α=0.05. We used a one sample proportion test (R function: *pwr.p.test {pwr}*) and obtained *n*=47 tumor samples. Importantly, this analysis does not depend on the number of marker genes assessed. Next, for training and two validation sets we calculated effect sizes for multiple power levels: 0.80, 0.95, and 0.99. Given that cases and controls were not matched, we used unpaired t-test to calculate effect sizes (R function: *pwr.t2n.test {pwr}*).

| **Set** | **N_Control_** | **N_Tumor_** | **Effect Size (Power=0.8)** | **Effect Size (Power=0.95)** | **Effect Size (Power=0.99)** |
| --- | --- | --- | --- | --- | --- |
| Training | 67 | 63 | 0.5 | 0.64 | 0.76 |
| Validation 1 | 43 | 72 | 0.54 | 0.7 | 0.83 |
| Validation 2 | 49 | 71 | 0.52 | 0.68 | 0.8 |

For each sample, effect sizes were ≥0.5, therefore we considered them acceptable [[40](#_ENREF_40)].

**Real-time PCR analysis of peripheral blood gene expression**

Real-time PCR was performed (384-well plate, HT-7900 machine) with 200ng/ul of cDNA and 16ul of reagents/well (Fast Universal PCR master mix, Applied Biosystems). PCR values were normalized to *ALG9* (ΔΔC_T_) [[44](#_ENREF_44)], using the control group as the population control (calibrator sample). All primers used were exon spanning and were <160bprs.

The limit of detection for PCR was 40 cycles (200ng/ul cDNA amplified: 95.3±0.2% of cases). Increasing the number of cycles to 45-50 cycles identified positive expression in <1% of target samples; the false negative rate using a C_T_ cut-off of 40 was 0.8%. This is more stringent than for leukemia detection [[45](#_ENREF_45)], but is consistent with other PCR-based detection protocols [[46-48](#_ENREF_46)]. A 2-step protocol (RNA isolation, cDNA production and PCR) was used (Pearson’s correlation 0.987-0.996).

**Blood-based PCR test – “majority” vote and generation of ROC curves**

The MATLAB algorithm generates the binary classification of “control” or “GEP-NEN”. Subsequently, a score of 0 was assigned to classifiers that predicted a sample as “control” (internally labeled as “N” reflecting normal), while a score of 1 was assigned to classifiers that predicted a sample to be “GEP-NEN” (internally labeled as “T” reflecting tumor). Therefore, we used the following rules to generate both the majority vote as well as the ROC curves.

If 4 “controls”, and 0 “GEP-NEN”; assign N=0 and T=0; total score = 0

If 3 “controls” and 1 “GEP-NEN”; assign N=0 and T=1; total score = 1

If 2 “controls” and 2 “GEP-NENs”; assign N=0 and T=1; total score= 2

If 1 “control” and 3 “GEP-NENs”; assign N=0 and T=1; total score = 3

If 0 “control” and 4 “GEP-NENs”; assign N=0 and T=1; total score = 4

If score < 2, sample = “control”, in cases where the Score > 2, the sample = “GEP-NEN”. Thus, in an event of a tie, the total score would be 2 and the majority vote would designate this sample as “Tumor”.

The “whole” or additive scores of 0-4 were then used to generate the ROC curves [[49](#_ENREF_49)]. Specifically, ROC curves were calculated using control and GEP-NEN samples as the independent variables versus the score (ranging from 0-4) which is the measurement of interest. The score as such provide five categories which yield non-trivial points on the ROC curve [[50](#_ENREF_50)].

**Supplementary Results**

**Gene co-expression network inference in GEP-NENs**

For each preprocessed microarray dataset (*n*=9, totaling 551 arrays) [[44](#_ENREF_44),[51-58](#_ENREF_51)], we calculated Pearson correlation coefficient (PCC) matrices corresponding to all pairwise gene expressions. It is clear that the size of a co-expression network depends on the threshold level selected. At low PCC thresholds, networks may be too large to be biologically informative, while at higher thresholds the networks will consist of a smaller number of genes and therefore may miss potentially interesting relationships. To systematically eliminate weak co-expressions for each dataset, a data-driven algorithm [[10](#_ENREF_10)] was implemented. The precise details of the algorithm we used are described in the methods above (**Pg 2**). PCC thresholds for GEP-NEN-A, GEP-NEN-B, Normal, Prostate, Breast, Colon, Viral-HCC, Alcohol-HCC, Progression-HCC datasets were 0.61, 0.96, 0.77, 0.59, 0.66, 0.75, 0.73, 0.76, and 0.65 respectively (**Figure S1**).

**
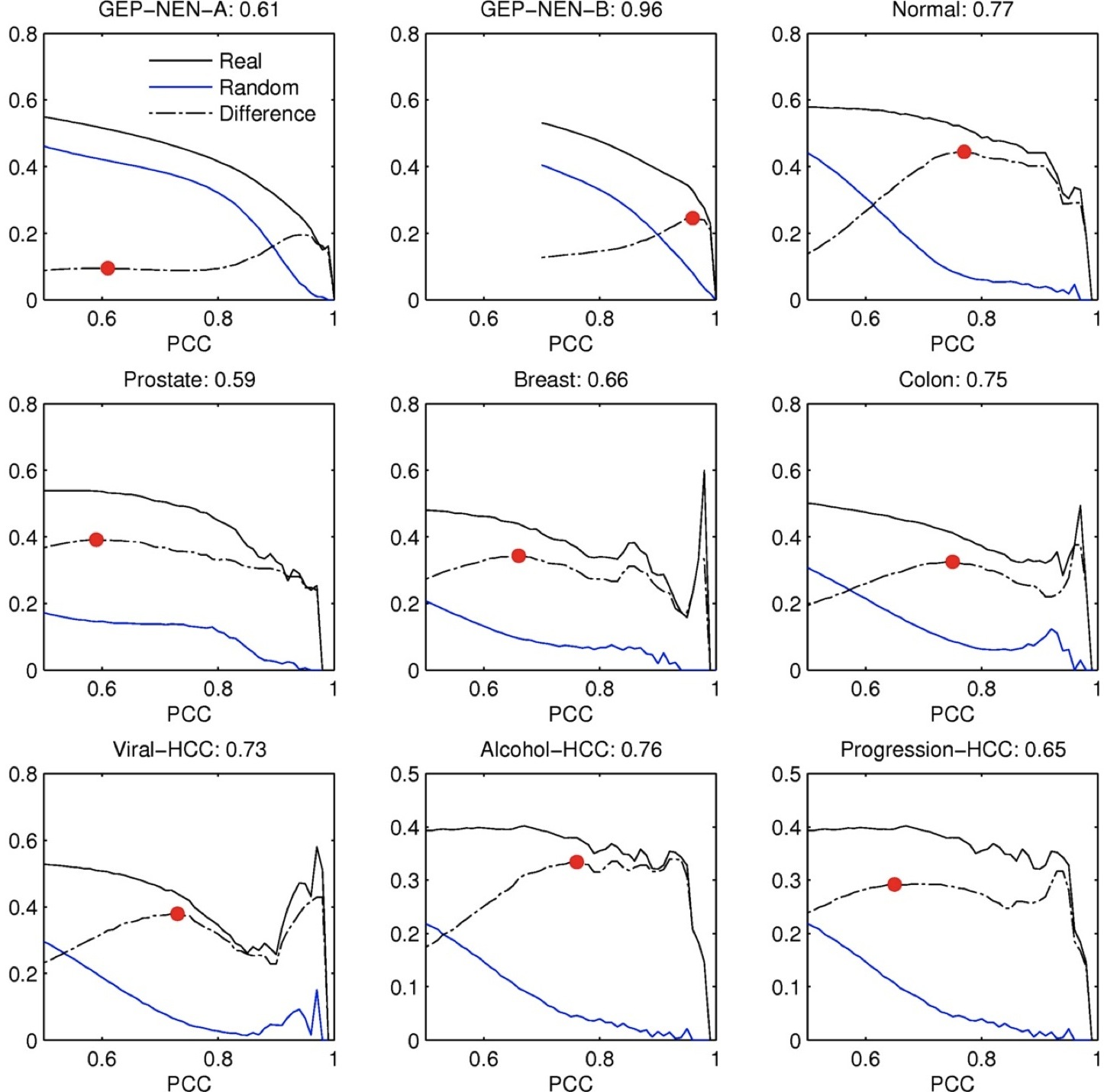
**

**Figure S1. Selection of Pearson correlation coefficient thresholds for gene co-expression network inference.** Clustering coefficients of real (black) and random (blue) networks with identical node degree distributions were systematically measured for 0.50≤PCC≤1.0. The threshold was selected at the first local maximum of the difference (red) between the real and random clustering coefficients. Because GEP-NEN-B networks were overly large, a PCC threshold was applied in the range of [0.70 1.0]. Calculated PCC threshold are shown as red points for each dataset.

Application of respective thresholds to the GEP-NEN-A-Net and GEP-NEN-B-Net resulted in 13110 genes and 13098820 links and 9745 genes and 244023 links, respectively. Intersection of these networks produced 3908 genes and 35944 links (*Step 1*, **Figure S2**). Taken together, other neoplastic and normal tissue networks contained 20184 genes and 3966629 links (*Step 2*, **Figure S2**). Overall, 3702 genes and 32576 links were specific to the GEP-NEN network (*Step 3*, **Figure S2**). Finally, we eliminated all genes from the GEP-NEN network that showed divergent regulation in tumor tissues compared to normal (*Step 4*, **Figure S2**), yielding 2545 genes and 30249 links (**Table S2**). This network was used further for enrichment and marker gene selection analysis.

**
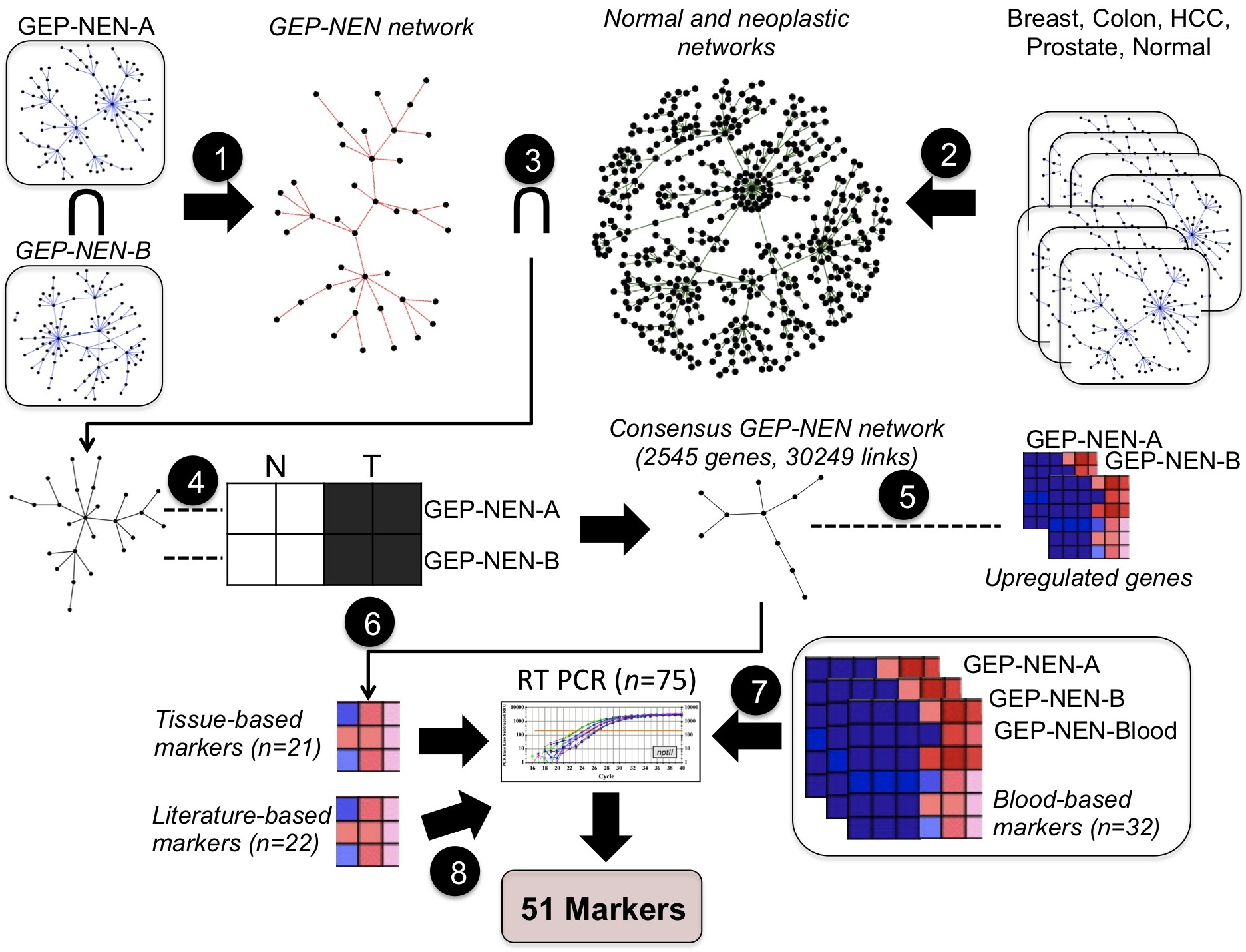
**

**Figure S2. Computational pipeline used to derive a set of 51 markers that identify GEP-NEN disease. Step 1:** Gene co-expression networks inferred from GEP-NEN-A and GEP-NEN-B datasets are intersected, producing the GEP-NEN network. **Step 2:** Co-expression networks from neoplastic and normal tissue microarray datasets are combined to produce the normal and neoplastic networks. **Step 3:** Links present in normal and neoplastic networks are subtracted from the GEP-NEN network. **Step 4:** Concordantly regulated genes in GEP-NEN-A and GEP-NEN-B networks are retained; other genes are eliminated from the GEP-NEN network, producing the Consensus GEP-NEN network. **Step 5:** Upregulated genes in both the GEP-NEN-A and GEP-NEN-B dataset are mapped to the Consensus GEP-NEN network. **Step 6:** Topological filtering, expression profiling, and literature-curation of putative tissue-based markers, yielding 21 putative genes further examined by RT-PCR. **Step 7:** Identification of mutually up-regulated genes in GEP-NEN blood transcriptome and GEP-NEN-A and GEP-NEN-B datasets, yielding 32 putative genes further examined by RT-PCR. **Step 8:** Literature-curation and cancer mutation database search, yielding a panel of 22 putative marker genes for further RT-PCR analysis.

**Marker gene selection**

*Tissue-based marker selection*

First, we aimed to generate a putative marker gene panel using tissue microarrays. Genes that were not present in the GEP-NEN network were removed from the GEP-NEN-A and GEP-NEN-B datasets. Subsequently, differential expression analysis, comparing tumor and normal tissue, was undertaken for both microarrays (*Step 5*, **Figure S2**). Genes with FDR corrected p-value < 0.05 were considered significant. There were 648 and 1154 significantly up-regulated genes in GEP-NEN-A and GEP-NEN-B datasets. Of these, 615 were up-regulated (FC>0) in both datasets and considered for further analysis. The large overlap (95% of GEP-NEN-A) of significantly up-regulated genes between the two datasets suggests that these two transcriptomes are comparable and contain reproducible changes in gene expression. To filter 615 genes further, we eliminated genes with fold changes in the lower 10^th^ percentile (FC < 0.72), adjusted p-values < 0.025, and co-expression network clustering coefficients < 0.25. We did not impose more strict differential expression thresholds because we aimed to expand the coverage of putative marker genes, thereby avoiding the loss of possibly biologically important hits. We integrated network clustering coefficient in the filtering process because high clustering coefficients were previously shown to be hallmarks of cancer-associated genes [[14](#_ENREF_14),[15](#_ENREF_15)]. This filtering approach produced 387 genes of which 18 did not have an assigned gene symbol. Therefore, we examined a set of 369 genes using a manual literature-curated search. Our search criteria involved implication of a putative marker gene in neuroendocrine axis as well as tumor formation or metastasis. Using these constraints, of the 369 putative genes, we selected 21 markers for further RT-PCR validation (*Step 6*, **Figure S2**).

*Blood-based marker selection*

We generated a transcriptome consisting of 14 peripheral blood samples (*n*=7 controls, *n*=7 GEP-NENs). We did not apply a stringent FC threshold, because it has previously been suggested that arbitrary FC cut offs may significantly alter microarray interpretation [[59](#_ENREF_59)]. Instead, differentially expressed genes were first ﬁltered for statistical signiﬁcance using the FDR correction, with an adjusted p-value cutoff of 0.05. However, this cutoff produced only 218 significantly up-regulated (FC>0) genes. Given that we focused only on up-regulated genes in our RT-PCR validation, we deemed this coverage to be low (1% of total genes), as a number of possibly important markers could have been missed [[59](#_ENREF_59)]. To expand the coverage of identified markers, we therefore used the raw p-values to rank genes by likelihood of differential expression. Reversion to raw p-values has previously been performed in similar analyses with a relatively low sample size [[59](#_ENREF_59)]. There were 1510 significantly up-regulated genes (*p*<0.05, FC>0) in GEP-NEN blood compared to healthy blood transcriptome. Of these, 1382 genes had an assigned gene symbol. Interestingly, the maximum fold change of differentially expressed genes in peripheral blood was 1.9, a substantially lower change compared to tissue samples (maximal FC=7). We filtered this list further and removed all genes with expression values in the lower 25^th^ quantile. This step was performed to capture only highly abundant transcripts. Additionally, we retained only those genes with positive FC in both tissue datasets (*Step 7*, **Figure S2**). This analysis produced 306 putative targets. Similarly to our approach for tissue-based target prioritization, we examined these genes using manual literature-curated search focusing on neoplasia-relevant hits. From this, 32/306 putative targets were selected for subsequent RT-PCR validation.

*Literature-based marker selection*

We carried out an extensive literature search and query of the Catalogue of Somatic Mutations in Cancer (COSMIC v60) database [[60](#_ENREF_60)] (*Step 8*, **Figure S2**) and identified thirteen marker genes that have been previously implicated in GEP-NENs, either in our studies [[61](#_ENREF_61),[62](#_ENREF_62)], or in others [[63-66](#_ENREF_63)]. An additional 9 genes were included in the literature-curated panel given their known association with tumor initiation and metastasis [[67-71](#_ENREF_67)].

Overall, the break-down of genes identified from the 75 candidate genes was as follows:

**Minimum expression threshold (*n*=51)**

Tissue based = 10

Blood based = 19

Literature curated = 22

**Differential expression filters (*n*=27)**

Tissue based = 5

Blood based = 7

Literature curated = 15

All differentially expressed genes were used for classifiers, therefore individual genes were not singled out as being more or less important. All genes were equally weighted for classification purposes.

**Specifics regarding the Chromogranin A assay**

In preliminary studies, the efficacy of CgA measurements (using the DAKO kit) was evaluated in a large control group (*n*=36) which identified that 75% of samples exhibited levels <14U/L. Analysis of various cut-off values identified the recommended DAKO level of 19U/L resulted in a false positive of 3%. This cut-off was chosen for the current study. Results using the DAKO kit were also compared with those from clinical chemistry (Quest Diagnostics). In 28 GEP-NENs, the relationship between the two measurements was significant (R^2^=0.22, *p*=0.04, *linear regression*). Using QUEST, 8/28 (29%) of samples were elevated compared to 7/28 (25%) with the DAKO kit, with a concordance of 40% between the two kits i.e. both measured elevated CgAs from the same patient sample. These results are consistent with published data [[72](#_ENREF_72),[73](#_ENREF_73)], demonstrating the validity of using the DAKO kit as a comparator with the DAKO cut-off.

**Comparisons between ROC-generated AUCs**

We compared the area under the curves (AUCs) for each of the validations sets to the CgA curve (**Figure 2C – main document**) using the method of Hanley and McNeil [[74](#_ENREF_74)] which first calculates the standard error for each AUC, then the standard error of the differences between each of the AUCs and then the Z-statistic.

$$SE=\sqrt{\frac{A(1-A)+(n_{a}-1)(Q1-A^{2})+(n_{n}-1)(Q2-A^{2})}{n_{a}n_{n}}}$$

where *A* = AUC, *n_a_* and n_n_ are the number of abnormal/normal samples and *Q1* and *Q2* are estimated from:

$$Q1=\frac{A}{2-A}$$

and

$$Q2=\frac{2A^{2}}{1+A}$$

Using this approach the following data was calculated for:

CgA set, Validation Set 1 and Validation Set 2:

SE = 0.04198; 0.012114; 0.019454

To calculate the SE of the differences between two AUCs, this was calculated by:

$$SE(A1-A2)=\sqrt{SE^{2}(A1)+SE^{2}(A2)-2rSE(A1)SE(A2)}$$

Using this approach (which was chosen because samples overlap between the CgA test set and each of the validation sets (*n*=29 for Validation Set 1 [n=15 NENs, n=14 controls[, *n*=16 [n=9 NENs, n=7 controls] for Validation Set 2), the *r*-value was derived from Table 1 [[75](#_ENREF_75)]) the following data was calculated for the differences between:

CgA set and Validation Set 1 and CgA set and Validation Set 2:

ΔSE = 0.032162 and 0.027131 (high r-value >0.8)

ΔSE = 0.042513 and 0.044468 (low r-value >0.1)

The Z-statistic was calculated by:

$$z=\frac{A1-A2}{SE(A1-A2)}$$

Using this method the following Z-values were calculated for:

CgA set vs. Validation Set 1 and CgA set vs. Validation Set 2:

Ζ = 10.57151 and 11.42601 (high r-value)

Ζ = 7.997504 and 6.971228 (low r-value)

We used a cut-off for Ζ of 1.96 (5%, Type I error).

Ζ values for each of these analyses (irrespective of the overlap between sample sets) are highly significant and identify the curves are significantly different.

**References**

1. Parkinson H, Kapushesky M, Kolesnikov N, Rustici G, Shojatalab M, et al. (2009) ArrayExpress update--from an archive of functional genomics experiments to the atlas of gene expression. Nucleic Acids Res 37: D868-872.

2. Irizarry RA, Hobbs B, Collin F, Beazer-Barclay YD, Antonellis KJ, et al. (2003) Exploration, normalization, and summaries of high density oligonucleotide array probe level data. Biostatistics 4: 249-264.

3. Gautier L, Cope L, Bolstad BM, Irizarry RA (2004) affy--analysis of Affymetrix GeneChip data at the probe level. Bioinformatics 20: 307-315.

4. Hubbell E, Liu WM, Mei R (2002) Robust estimators for expression analysis. Bioinformatics 18: 1585-1592.

5. Li C, Wong WH (2001) Model-based analysis of oligonucleotide arrays: expression index computation and outlier detection. Proceedings of the National Academy of Sciences of the United States of America 98: 31-36.

6. Irizarry RA, Bolstad BM, Collin F, Cope LM, Hobbs B, et al. (2003) Summaries of Affymetrix GeneChip probe level data. Nucleic acids research 31: e15.

7. Stalteri MA, Harrison AP (2007) Interpretation of multiple probe sets mapping to the same gene in Affymetrix GeneChips. BMC Bioinformatics 8: 13.

8. Sproul D, Nestor C, Culley J, Dickson JH, Dixon JM, et al. (2011) Transcriptionally repressed genes become aberrantly methylated and distinguish tumors of different lineages in breast cancer. Proceedings of the National Academy of Sciences of the United States of America 108: 4364-4369.

9. Birney E, Andrews TD, Bevan P, Caccamo M, Chen Y, et al. (2004) An overview of Ensembl. Genome Res 14: 925-928.

10. Elo LL, Jarvenpaa H, Oresic M, Lahesmaa R, Aittokallio T (2007) Systematic construction of gene coexpression networks with applications to human T helper cell differentiation process. Bioinformatics 23: 2096-2103.

11. Maslov S, Sneppen K (2002) Specificity and stability in topology of protein networks. Science 296: 910-913.

12. Drozdov I, Bornschein J, Wex T, Valeyev NV, Tsoka S, et al. (2012) Functional and topological properties in hepatocellular carcinoma transcriptome. PloS one 7: e35510.

13. Feldman I, Rzhetsky A, Vitkup D (2008) Network properties of genes harboring inherited disease mutations. Proceedings of the National Academy of Sciences of the United States of America 105: 4323-4328.

14. Li L, Zhang K, Lee J, Cordes S, Davis DP, et al. (2009) Discovering cancer genes by integrating network and functional properties. BMC medical genomics 2: 61.

15. Kar G, Gursoy A, Keskin O (2009) Human cancer protein-protein interaction network: a structural perspective. PLoS computational biology 5: e1000601.

16. Blondel VD, Guillaume J-L, Lambiotte R, Lefebvre E (2008) Fast unfolding of communities in large networks. J Stat Mech P10008.

17. Newman ME (2006) Modularity and community structure in networks. Proceedings of the National Academy of Sciences of the United States of America 103: 8577-8582.

18. Xu G, Bennett L, Papageorgiou LG, Tsoka S (2010) Module detection in complex networks using integer optimisation. Algorithms for molecular biology : AMB 5: 36.

19. Drozdov I, Svejda B, Gustafsson BI, Mane S, Pfragner R, et al. (2011) Gene network inference and biochemical assessment delineates GPCR pathways and CREB targets in small intestinal neuroendocrine neoplasia. PloS one 6: e22457.

20. Meunier D, Lambiotte R, Fornito A, Ersche KD, Bullmore ET (2009) Hierarchical modularity in human brain functional networks. Frontiers in neuroinformatics 3: 37.

21. Haynes J, Perisic I (2010) Mapping search relevance to social networks. Proceedings of the 3rd Workshop on Social Network Mining and Analysis.

22. Parry RM, Jones W, Stokes TH, Phan JH, Moffitt RA, et al. (2010) k-Nearest neighbor models for microarray gene expression analysis and clinical outcome prediction. The pharmacogenomics journal 10: 292-309.

23. Drozdov I, Ouzounis CA, Shah AM, Tsoka S (2011) Functional Genomics Assistant (FUGA): a toolbox for the analysis of complex biological networks. BMC research notes 4: 462.

24. Blondel VD, Guillaume J-L, Lambiotte R, Lefebvre E (2008) Fast unfolding of communities in large network. J Stat Mech P10008.

25. Huang da W, Sherman BT, Lempicki RA (2009) Systematic and integrative analysis of large gene lists using DAVID bioinformatics resources. Nat Protoc 4: 44-57.

26. Smyth GK (2005) Limma: Linear Models for Microarray Data. In: R. Gentleman VC, S. Dudoit, R. Irizarry, W. Huber, editor. Bioinformatics and Computational Biology Solutions using R and Bioconductor. New York: Springer. pp. 397–420.

27. Glotsos D, Tohka J, Ravazoula P, Cavouras D, Nikiforidis G (2005) Automated diagnosis of brain tumours astrocytomas using probabilistic neural network clustering and support vector machines. Int J Neural Syst 15: 1-11.

28. Mattfeldt T, Gottfried HW, Wolter H, Schmidt V, Kestler HA, et al. (2003) Classification of prostatic carcinoma with artificial neural networks using comparative genomic hybridization and quantitative stereological data. Pathol Res Pract 199: 773-784.

29. Zander T, Hofmann A, Staratschek-Jox A, Classen S, Debey-Pascher S, et al. (2011) Blood-based gene expression signatures in non-small cell lung cancer. Clinical cancer research : an official journal of the American Association for Cancer Research 17: 3360-3367.

30. De Ferrari L, Aitken S (2006) Mining housekeeping genes with a Naive Bayes classifier. BMC Genomics 7: 277.

31. Demsar J, Zupan B, Kattan MW, Beck JR, Bratko I (1999) Naive Bayesian-based nomogram for prediction of prostate cancer recurrence. Studies in health technology and informatics 68: 436-441.

32. Bosman FT (2010) WHO classification of tumours of the digestive system. Lyon: World Health Organization.; International Agency for Research on Cancer.

33. Lawrence B, Gustafsson BI, Chan A, Svejda B, Kidd M, et al. (2011) The epidemiology of gastroenteropancreatic neuroendocrine tumors. Endocrinol Metab Clin North Am 40: 1-18, vii.

34. Yao JC, Hassan M, Phan A, Dagohoy C, Leary C, et al. (2008) One hundred years after "carcinoid": epidemiology of and prognostic factors for neuroendocrine tumors in 35,825 cases in the United States. J Clin Oncol 26: 3063-3072.

35. Raza A, Ali Z, Irfan J, Murtaza S, Shakeel S (2012) Analytical variables influencing the HCV RNA determination by TaqMan real-time PCR in routine clinical laboratory practice. Mol Biol Rep 39: 7421-7427. Epub 2012 Feb 7412.

36. Kelsey J, Whittemore A, Evans A, Thompson W (1996) Methods in Observational Epidemiology. . New York, NY: : Oxford University Press.

37. Fleiss J, Levin B, Paik M (2004) Statistical Methods for Rates and Proportions, Third Edition. In: Shewart W, Wilks S, editors. Wiley Series in Probability and Statistics: Wiley.

38. Luther C, Wienhold W, Oehlmann R, Heinemann MK, Melms A, et al. (2005) Alternatively spliced transcripts of the thymus-specific protease PRSS16 are differentially expressed in human thymus. Genes Immun 6: 1-7.

39. Chervoneva I, Li Y, Schulz S, Croker S, Wilson C, et al. (2010) Selection of optimal reference genes for normalization in quantitative RT-PCR. BMC Bioinformatics 11: 253.

40. Cohen J (1988) Statistical power analysis for the behavioral sciences; Erlbaum L, editor. Hillsdale, NJ.

41. O'Bryant SE, Xiao G, Barber R, Reisch J, Doody R, et al. (2010) A serum protein-based algorithm for the detection of Alzheimer disease. Archives of neurology 67: 1077-1081.

42. Biliavska I, Stamm TA, Martinez-Avila J, Huizinga TW, Landewe RB, et al. (2012) Application of the 2010 ACR/EULAR classification criteria in patients with very early inflammatory arthritis: analysis of sensitivity, specificity and predictive values in the SAVE study cohort. Annals of the rheumatic diseases.

43. Majewski T, Spiess PE, Bondaruk J, Black P, Clarke C, et al. (2012) Detection of bladder cancer using proteomic profiling of urine sediments. PloS one 7: e42452.

44. Kidd M, Nadler B, Mane S, Eick G, Malfertheiner M, et al. (2007) GeneChip, geNorm, and gastrointestinal tumors: novel reference genes for real-time PCR. Physiological genomics 30: 363-370.

45. Gabert J, Beillard E, van der Velden VH, Bi W, Grimwade D, et al. (2003) Standardization and quality control studies of 'real-time' quantitative reverse transcriptase polymerase chain reaction of fusion gene transcripts for residual disease detection in leukemia - a Europe Against Cancer program. Leukemia 17: 2318-2357.

46. Hur M, Moon HW, Yun YM, Kang TY, Kim HS, et al. (2011) Detection of tuberculosis using artus M. tuberculosis PCR Kit and COBAS AMPLICOR Mycobacterium tuberculosis Test. Int J Tuberc Lung Dis 15: 795-798.

47. Merker JD, Jones CD, Oh ST, Schrijver I, Gotlib J, et al. (2010) Design and evaluation of a real-time PCR assay for quantification of JAK2 V617F and wild-type JAK2 transcript levels in the clinical laboratory. J Mol Diagn 12: 58-64. Epub 2009 Dec 2003.

48. Burns MJ, Valdivia H, Harris N (2004) Analysis and interpretation of data from real-time PCR trace detection methods using quantitation of GM soya as a model system. Anal Bioanal Chem 378: 1616-1623.

49. Zweig MH, Campbell G (1993) Receiver-operating characteristic (ROC) plots: a fundamental evaluation tool in clinical medicine. Clin Chem 39: 561-577.

50. Metz CE, Shen JH (1992) Gains in accuracy from replicated readings of diagnostic images: prediction and assessment in terms of ROC analysis. Med Decis Making 12: 60-75.

51. Boyault S, Rickman DS, de Reynies A, Balabaud C, Rebouissou S, et al. (2007) Transcriptome classification of HCC is related to gene alterations and to new therapeutic targets. Hepatology 45: 42-52.

52. Mas VR, Maluf DG, Archer KJ, Yanek K, Williams B, et al. (2006) Differentially expressed genes between early and advanced hepatocellular carcinoma (HCC) as a potential tool for selecting liver transplant recipients. Mol Med 12: 97-104.

53. Wurmbach E, Chen YB, Khitrov G, Zhang W, Roayaie S, et al. (2007) Genome-wide molecular profiles of HCV-induced dysplasia and hepatocellular carcinoma. Hepatology 45: 938-947.

54. Pau Ni IB, Zakaria Z, Muhammad R, Abdullah N, Ibrahim N, et al. (2010) Gene expression patterns distinguish breast carcinomas from normal breast tissues: the Malaysian context. Pathol Res Pract 206: 223-228.

55. Ancona N, Maglietta R, Piepoli A, D'Addabbo A, Cotugno R, et al. (2006) On the statistical assessment of classifiers using DNA microarray data. BMC Bioinformatics 7: 387.

56. Koziol JA, Feng AC, Jia Z, Wang Y, Goodison S, et al. (2009) The wisdom of the commons: ensemble tree classifiers for prostate cancer prognosis. Bioinformatics 25: 54-60.

57. Su AI, Wiltshire T, Batalov S, Lapp H, Ching KA, et al. (2004) A gene atlas of the mouse and human protein-encoding transcriptomes. Proceedings of the National Academy of Sciences of the United States of America 101: 6062-6067.

58. Leja J, Essaghir A, Essand M, Wester K, Oberg K, et al. (2009) Novel markers for enterochromaffin cells and gastrointestinal neuroendocrine carcinomas. Mod Pathol 22: 261-272.

59. Dalman MR, Deeter A, Nimishakavi G, Duan ZH (2012) Fold change and p-value cutoffs significantly alter microarray interpretations. BMC Bioinformatics 13 Suppl 2: S11.

60. Forbes SA, Bindal N, Bamford S, Cole C, Kok CY, et al. (2011) COSMIC: mining complete cancer genomes in the Catalogue of Somatic Mutations in Cancer. Nucleic acids research 39: D945-950.

61. Drozdov I, Kidd M, Nadler B, Camp RL, Mane SM, et al. (2009) Predicting neuroendocrine tumor (carcinoid) neoplasia using gene expression profiling and supervised machine learning. Cancer 115: 1638-1650.

62. Kidd M, Modlin IM, Mane SM, Camp RL, Eick G, et al. (2006) The role of genetic markers--NAP1L1, MAGE-D2, and MTA1--in defining small-intestinal carcinoid neoplasia. Ann Surg Oncol 13: 253-262. Epub 2006 Jan 2020.

63. Cui T, Hurtig M, Elgue G, Li SC, Veronesi G, et al. (2010) Paraneoplastic antigen Ma2 autoantibodies as specific blood biomarkers for detection of early recurrence of small intestine neuroendocrine tumors. PLoS One 5: e16010.

64. Karhoff D, Sauer S, Schrader J, Arnold R, Fendrich V, et al. (2007) Rap1/B-Raf signaling is activated in neuroendocrine tumors of the digestive tract and Raf kinase inhibition constitutes a putative therapeutic target. Neuroendocrinology 85: 45-53.

65. Wulbrand U, Wied M, Zofel P, Goke B, Arnold R, et al. (1998) Growth factor receptor expression in human gastroenteropancreatic neuroendocrine tumours. Eur J Clin Invest 28: 1038-1049.

66. Muscarella LA, D'Alessandro V, la Torre A, Copetti M, De Cata A, et al. (2011) Gene expression of somatostatin receptor subtypes SSTR2a, SSTR3 and SSTR5 in peripheral blood of neuroendocrine lung cancer affected patients. Cell Oncol 19: 19.

67. Bralten LB, Kloosterhof NK, Gravendeel LA, Sacchetti A, Duijm EJ, et al. (2010) Integrated genomic profiling identifies candidate genes implicated in glioma-genesis and a novel LEO1-SLC12A1 fusion gene. Genes, chromosomes & cancer 49: 509-517.

68. Kim M, Jang HR, Kim JH, Noh SM, Song KS, et al. (2008) Epigenetic inactivation of protein kinase D1 in gastric cancer and its role in gastric cancer cell migration and invasion. Carcinogenesis 29: 629-637.

69. Miretti S, Roato I, Taulli R, Ponzetto C, Cilli M, et al. (2008) A mouse model of pulmonary metastasis from spontaneous osteosarcoma monitored in vivo by Luciferase imaging. PloS one 3: e1828.

70. Hogarty MD, Norris MD, Davis K, Liu X, Evageliou NF, et al. (2008) ODC1 is a critical determinant of MYCN oncogenesis and a therapeutic target in neuroblastoma. Cancer research 68: 9735-9745.

71. Naik S, Dothager RS, Marasa J, Lewis CL, Piwnica-Worms D (2009) Vascular Endothelial Growth Factor Receptor-1 Is Synthetic Lethal to Aberrant {beta}-Catenin Activation in Colon Cancer. Clinical cancer research : an official journal of the American Association for Cancer Research 15: 7529-7537.

72. Stridsberg M, Eriksson B, Oberg K, Janson ET (2003) A comparison between three commercial kits for chromogranin A measurements. J Endocrinol 177: 337-341.

73. Ramachandran R, Bech P, Murphy KG, Dhillo WS, Meeran KM, et al. (2012) Improved diagnostic accuracy for neuroendocrine neoplasms using two chromogranin A assays. Clin Endocrinol (Oxf) 76: 831-836. doi: 810.1111/j.1365-2265.2011.04319.x.

74. Hanley JA, McNeil BJ (1982) The meaning and use of the area under a receiver operating characteristic (ROC) curve. Radiology 143: 29-36.

75. Hanley JA, McNeil BJ (1983) A method of comparing the areas under receiver operating characteristic curves derived from the same cases. Radiology 148: 839-843.
